# Supplementary figures and images for: Identification of p38 MAPK inhibition as a neuroprotective strategy for combinatorial SMA therapy
Source: EMBO Mol Med. 2025 Sep 8;17(10):2762–86. doi: 10.1038/s44321-025-00303-6 (PMC12514318; doi:10.1038/s44321-025-00303-6)

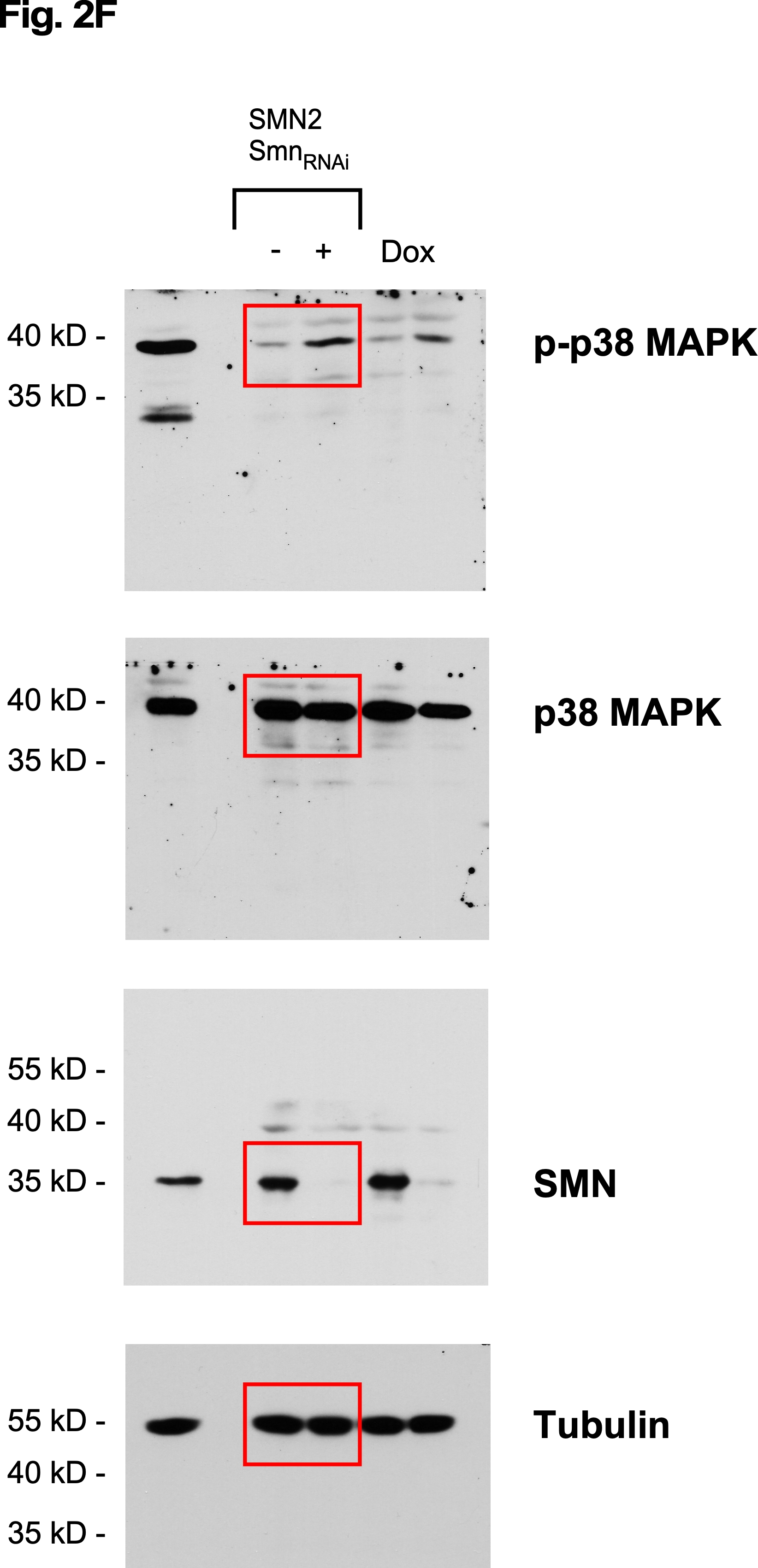

Supplement: Supplementary file 5 — Source data Fig. 2 [file 44321_2025_303_MOESM5_ESM.zip › Figure 2/2F/2F_WB.jpg]

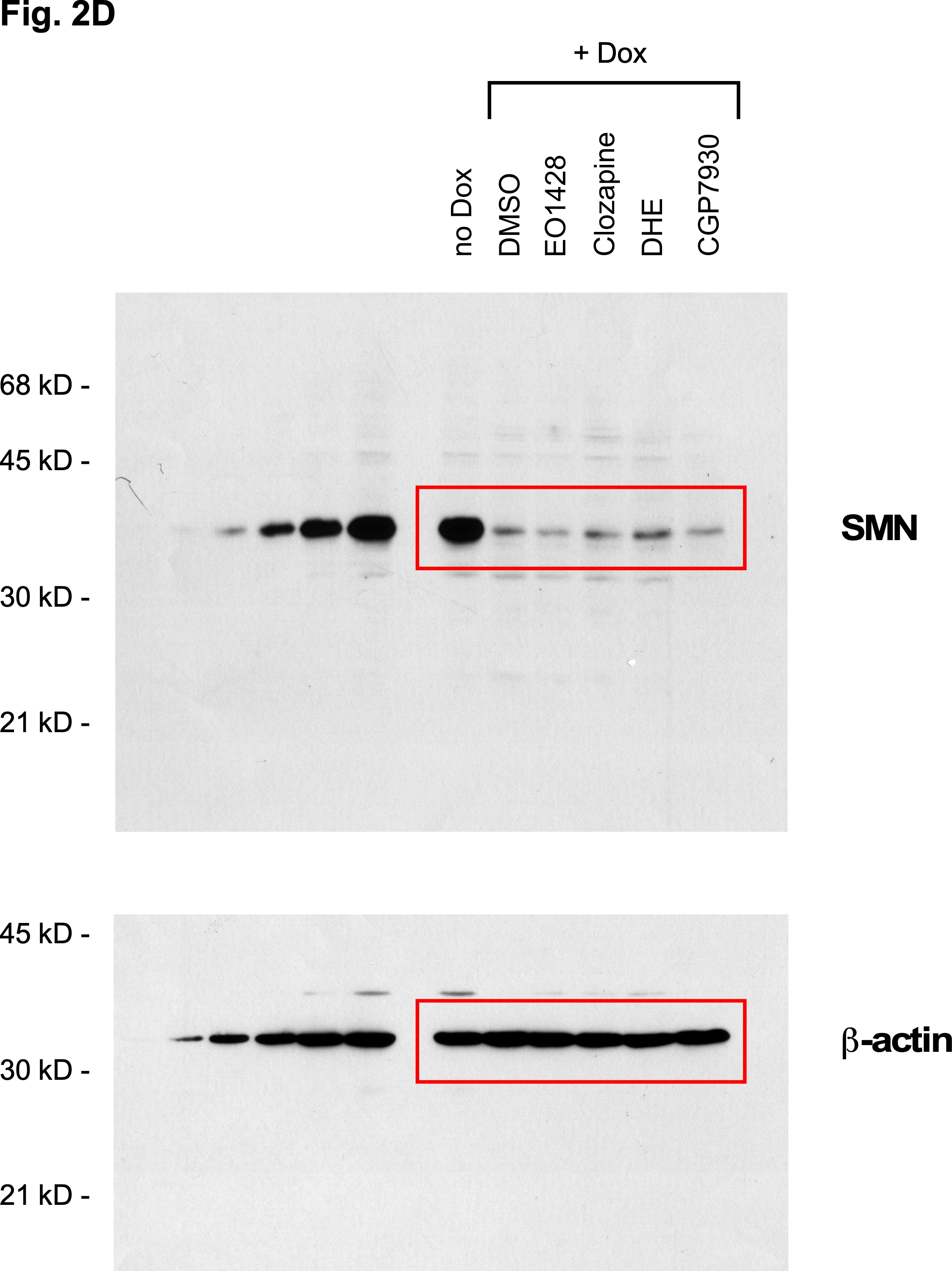

Supplement: Supplementary file 5 — Source data Fig. 2 [file 44321_2025_303_MOESM5_ESM.zip › Figure 2/2D/2D_WB.jpg]

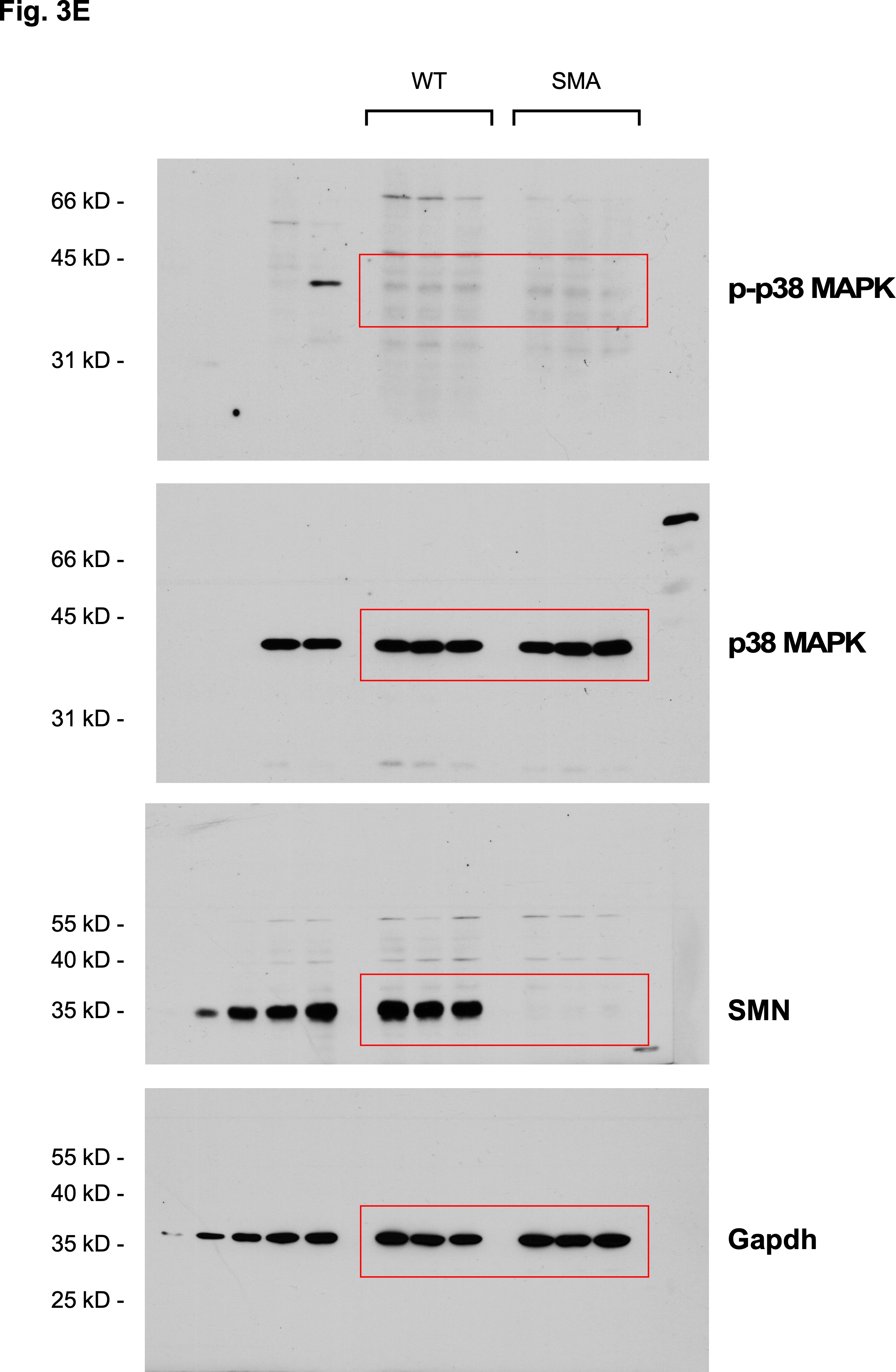

Supplement: Supplementary file 6 — Source data Fig. 3 [file 44321_2025_303_MOESM6_ESM.zip › Figure 3/3E/3E_WB.jpg]

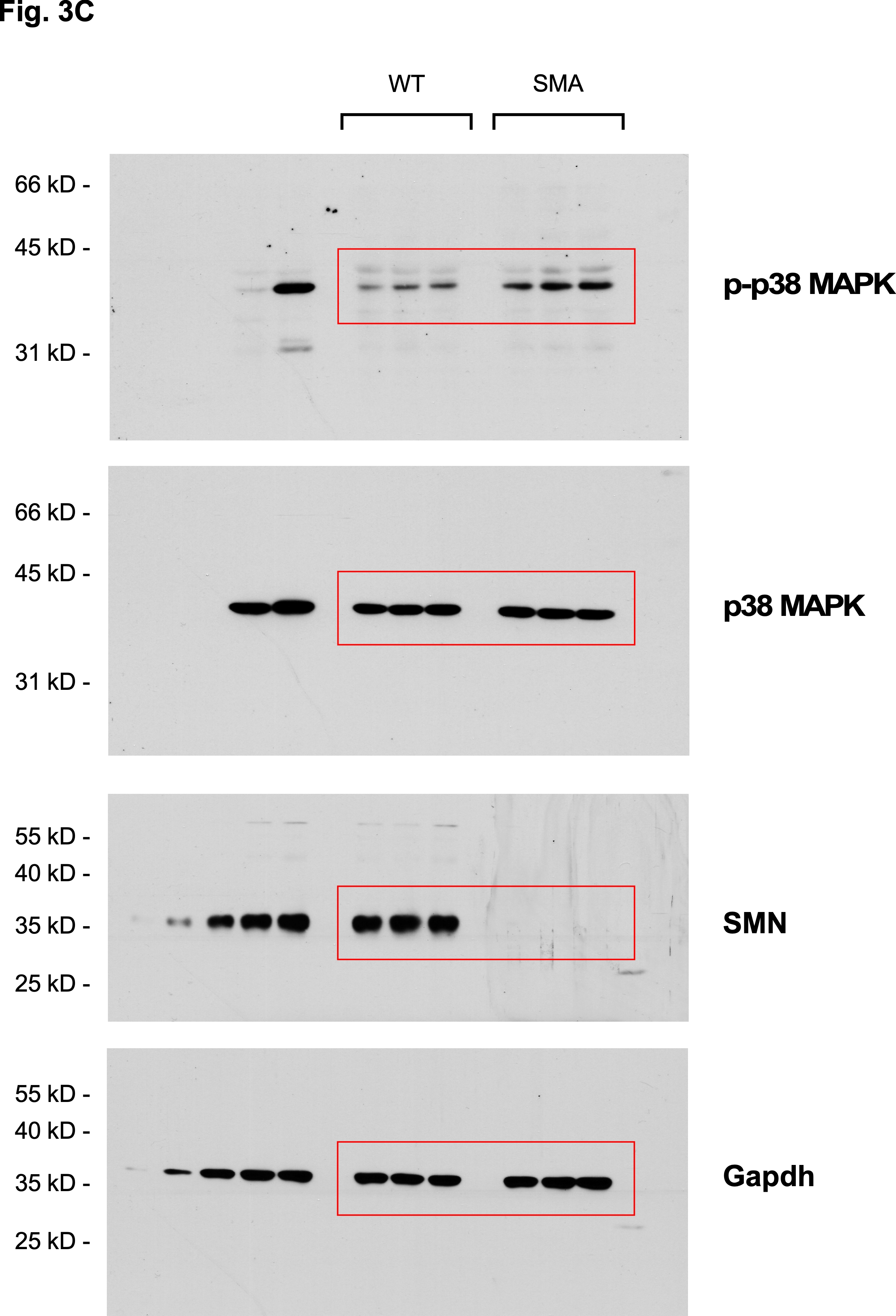

Supplement: Supplementary file 6 — Source data Fig. 3 [file 44321_2025_303_MOESM6_ESM.zip › Figure 3/3C/3C_WB.jpg]

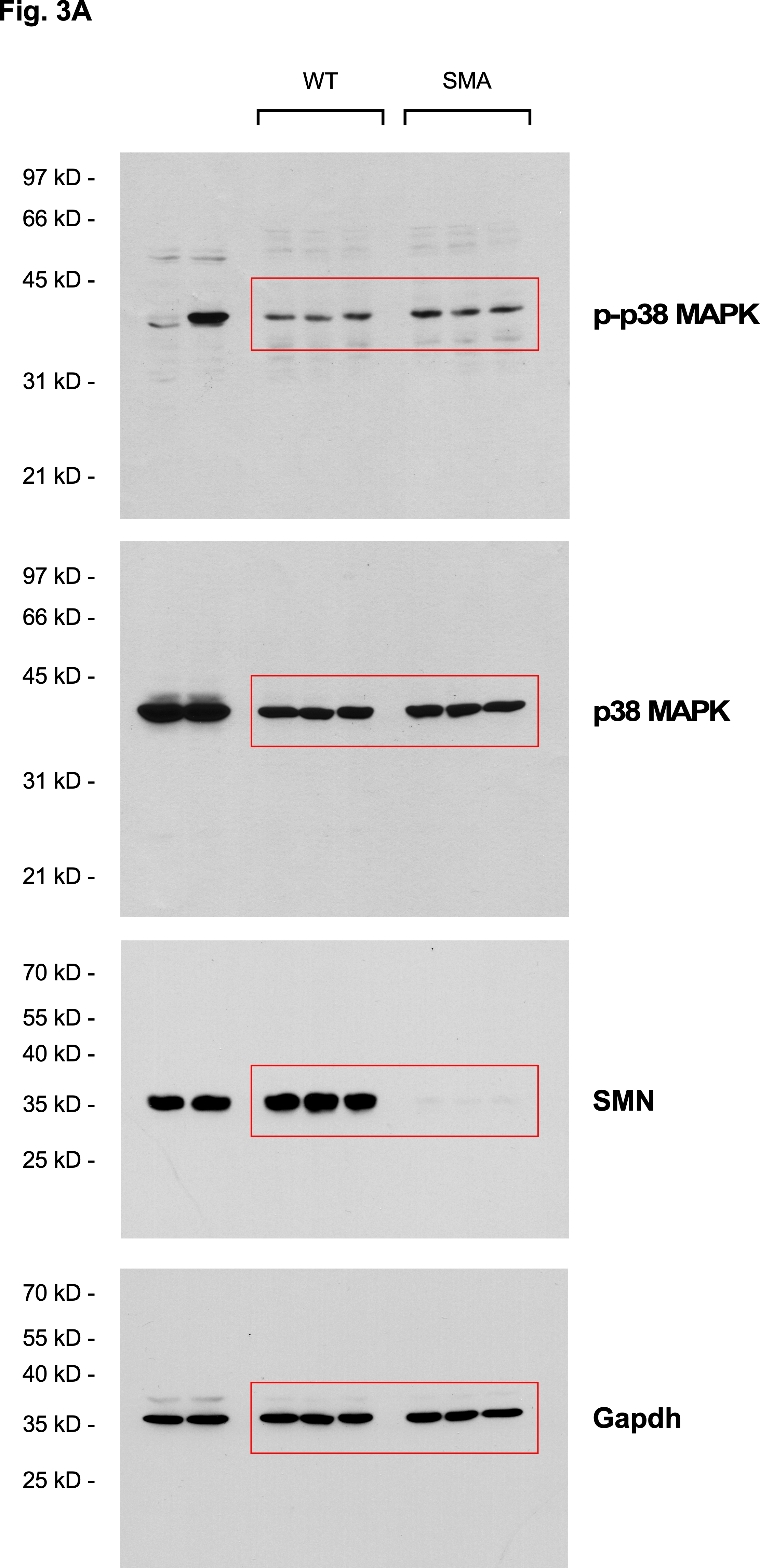

Supplement: Supplementary file 6 — Source data Fig. 3 [file 44321_2025_303_MOESM6_ESM.zip › Figure 3/3A/3A_WB.jpg]

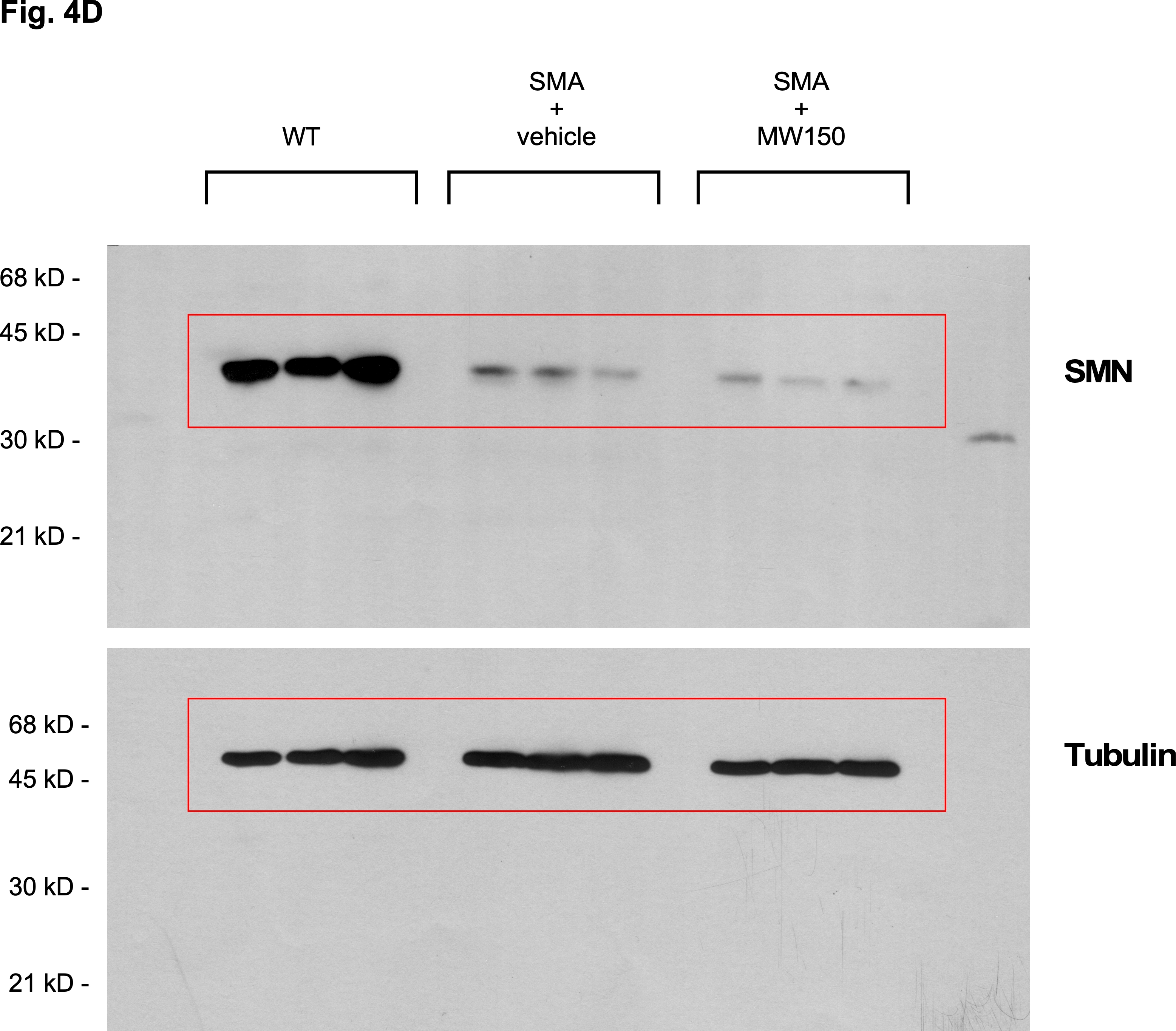

Supplement: Supplementary file 7 — Source data Fig. 4 [file 44321_2025_303_MOESM7_ESM.zip › Figure 4/4D/4D_WB.jpg]

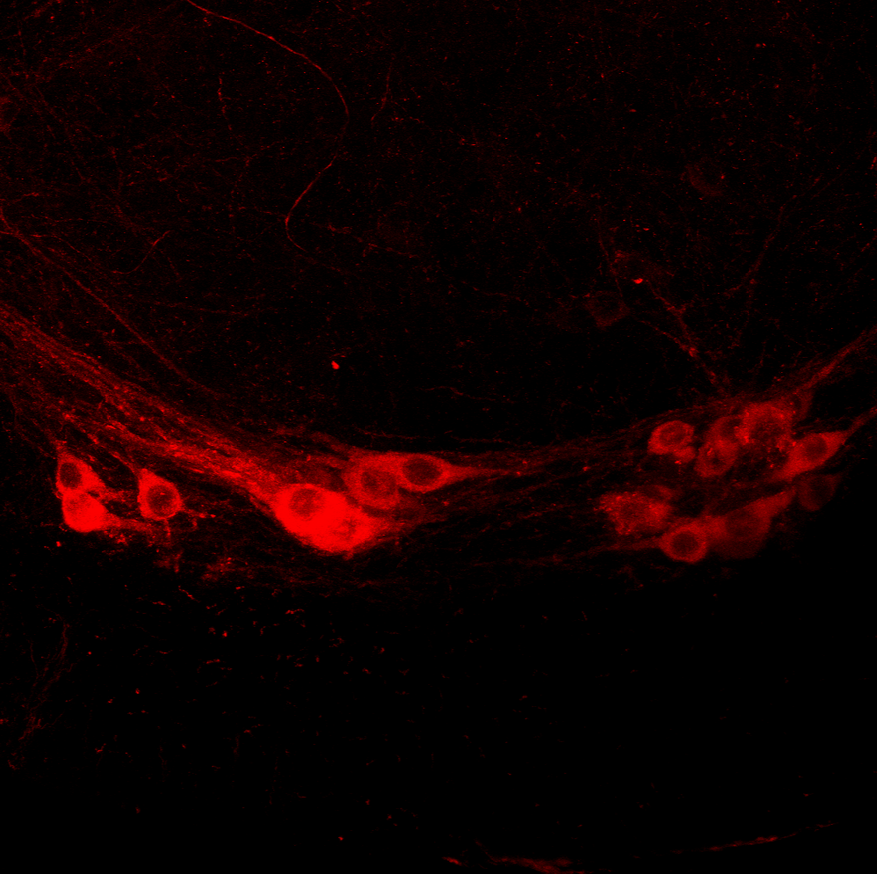

Supplement: Supplementary file 7 — Source data Fig. 4 [file 44321_2025_303_MOESM7_ESM.zip › Figure 4/4F/4F_L2_SMA+vehicle.tif]

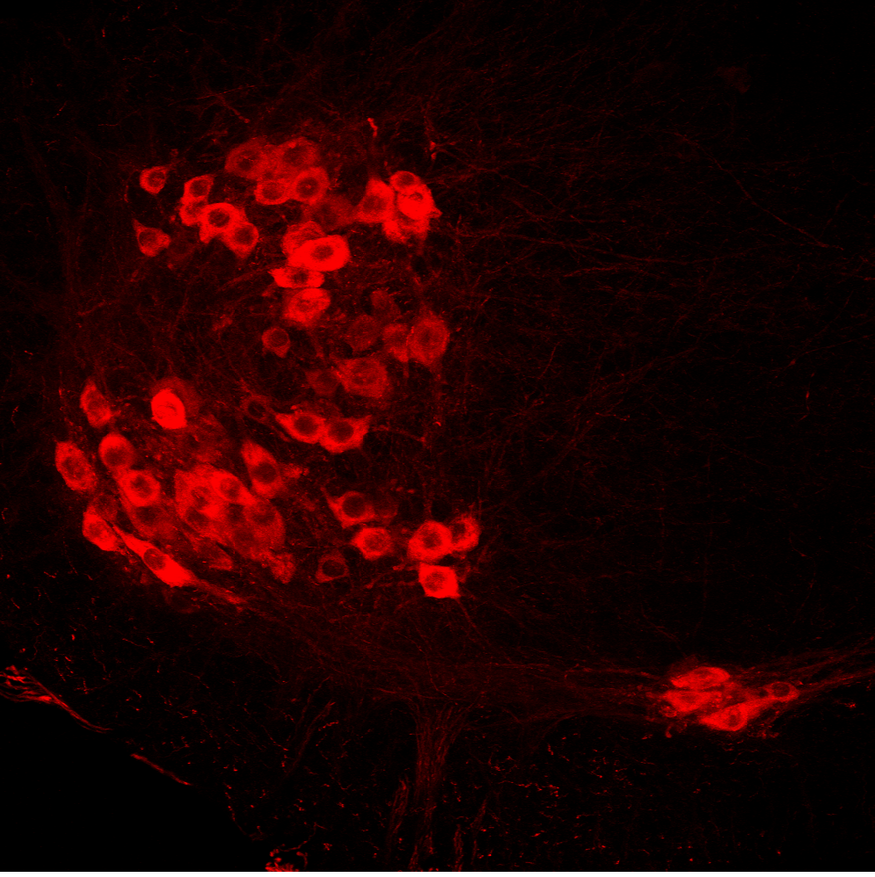

Supplement: Supplementary file 7 — Source data Fig. 4 [file 44321_2025_303_MOESM7_ESM.zip › Figure 4/4F/4F_L5_WT.tif]

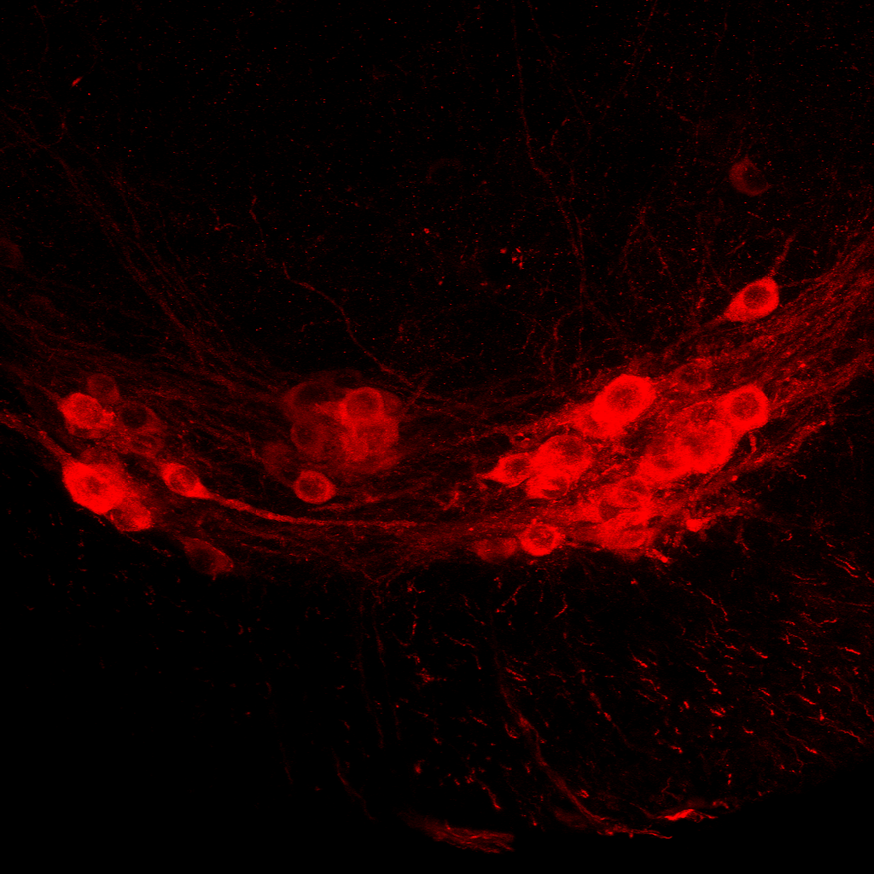

Supplement: Supplementary file 7 — Source data Fig. 4 [file 44321_2025_303_MOESM7_ESM.zip › Figure 4/4F/4F_L2_SMA+MW150.tif]

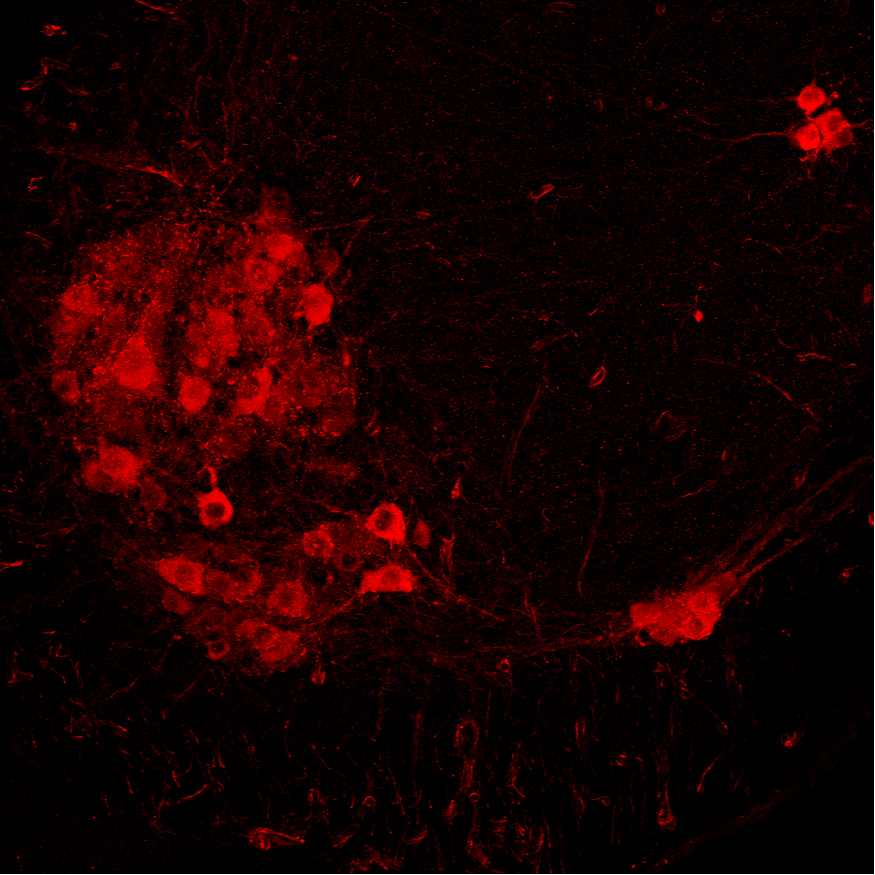

Supplement: Supplementary file 7 — Source data Fig. 4 [file 44321_2025_303_MOESM7_ESM.zip › Figure 4/4F/4F_L5_SMA+MW150.tif]

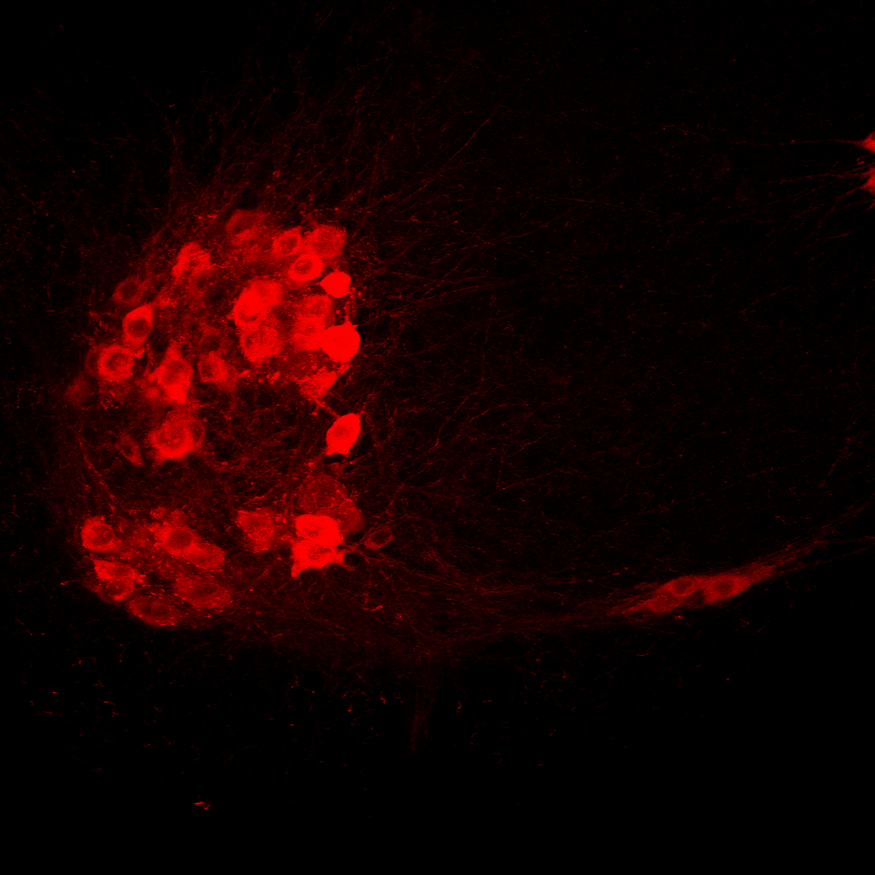

Supplement: Supplementary file 7 — Source data Fig. 4 [file 44321_2025_303_MOESM7_ESM.zip › Figure 4/4F/4F_L5_SMA+vehicle.tif]

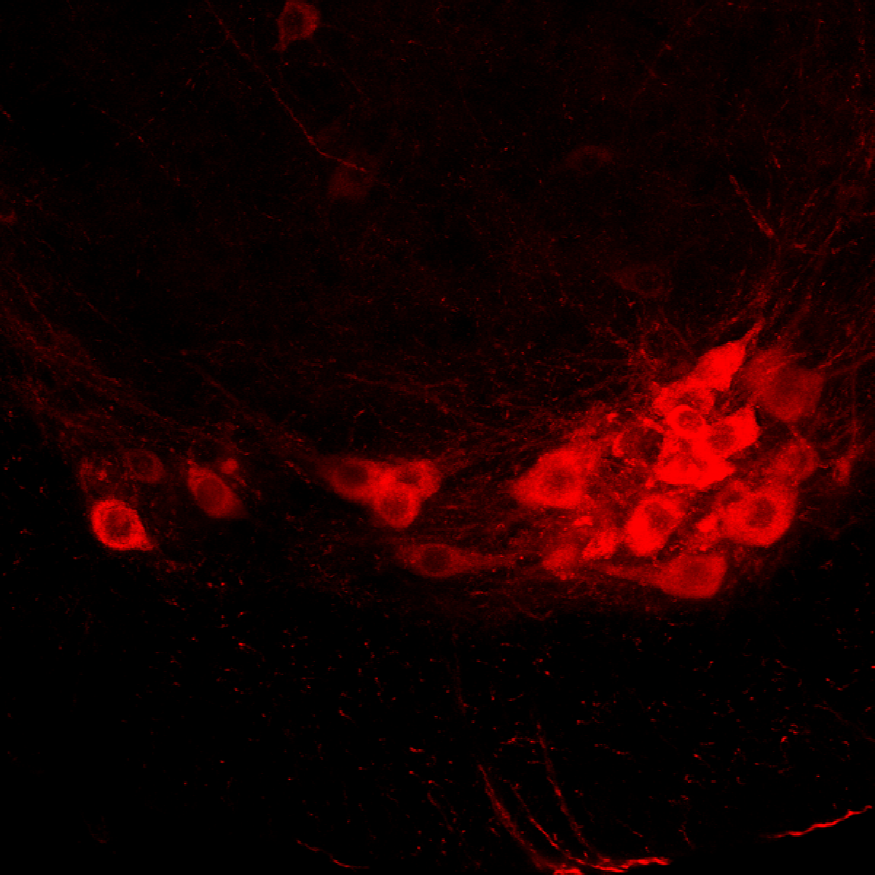

Supplement: Supplementary file 7 — Source data Fig. 4 [file 44321_2025_303_MOESM7_ESM.zip › Figure 4/4F/4F_L2_WT.tif]

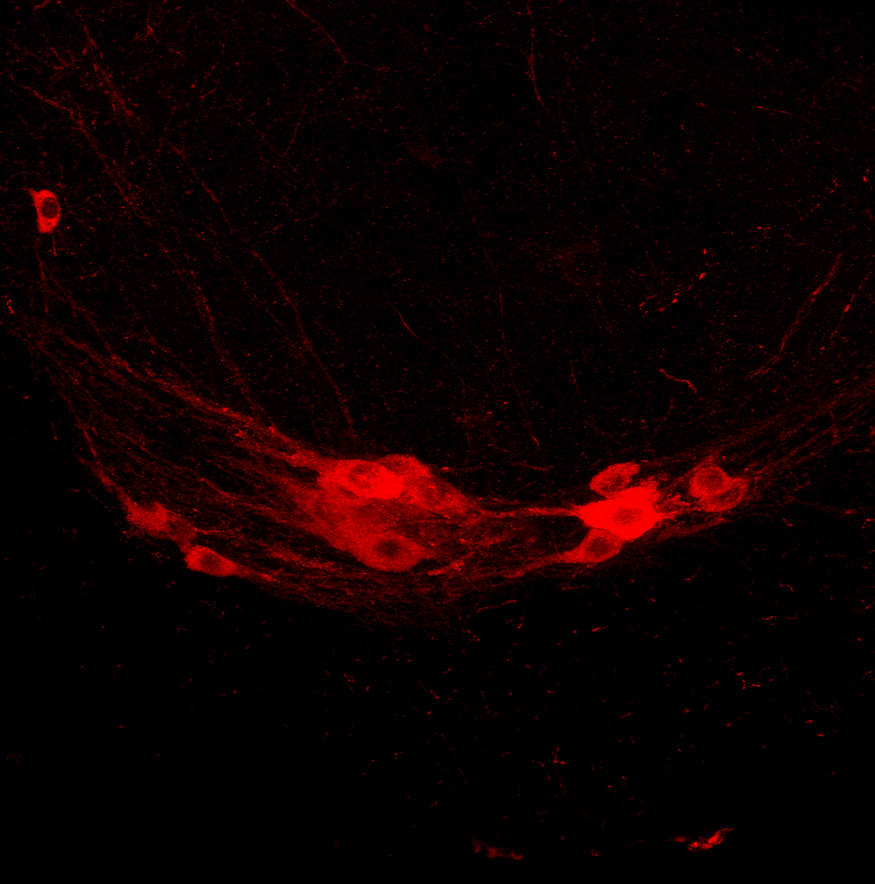

Supplement: Supplementary file 8 — Source data Fig. 5 [file 44321_2025_303_MOESM8_ESM.zip › Figure 5/5G/5G_L2_SMA+SMNC3(P8).tif]

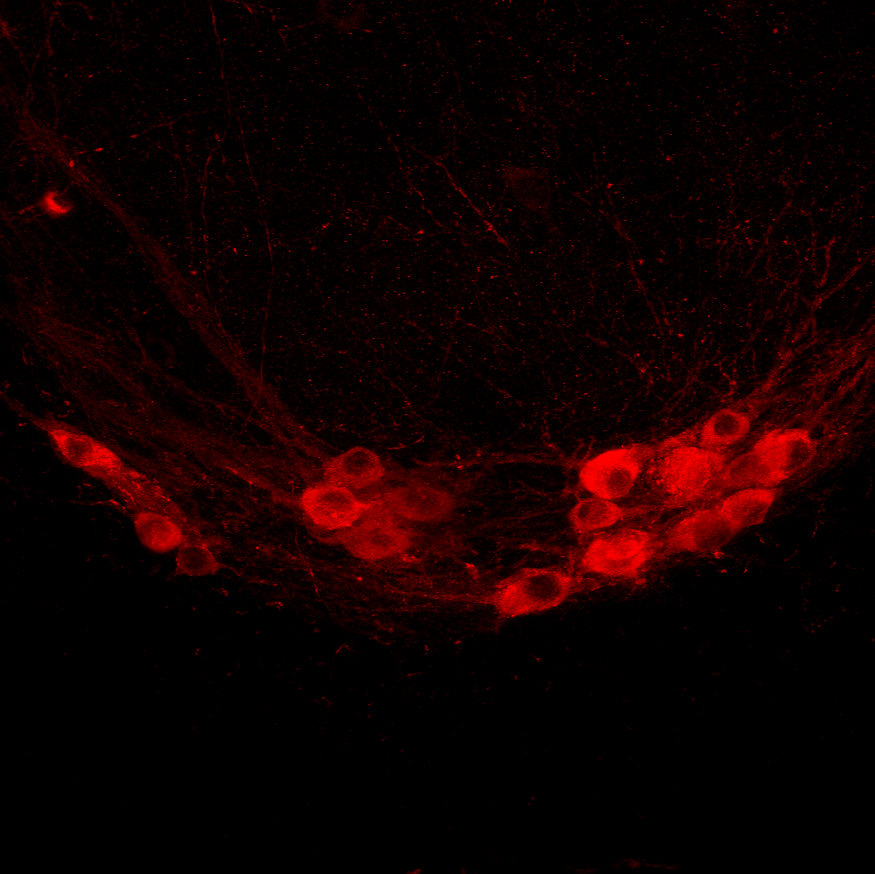

Supplement: Supplementary file 8 — Source data Fig. 5 [file 44321_2025_303_MOESM8_ESM.zip › Figure 5/5G/5G_L2_WT.tif]

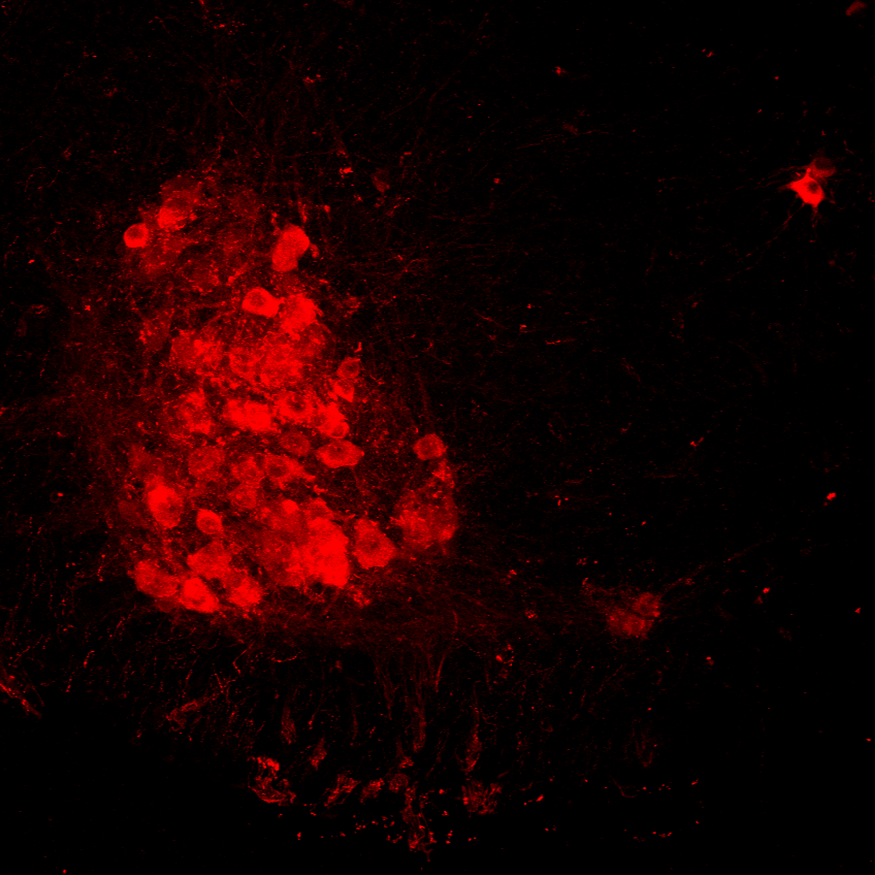

Supplement: Supplementary file 8 — Source data Fig. 5 [file 44321_2025_303_MOESM8_ESM.zip › Figure 5/5G/5G_L5_SMA+SMNC3(P8).tif]

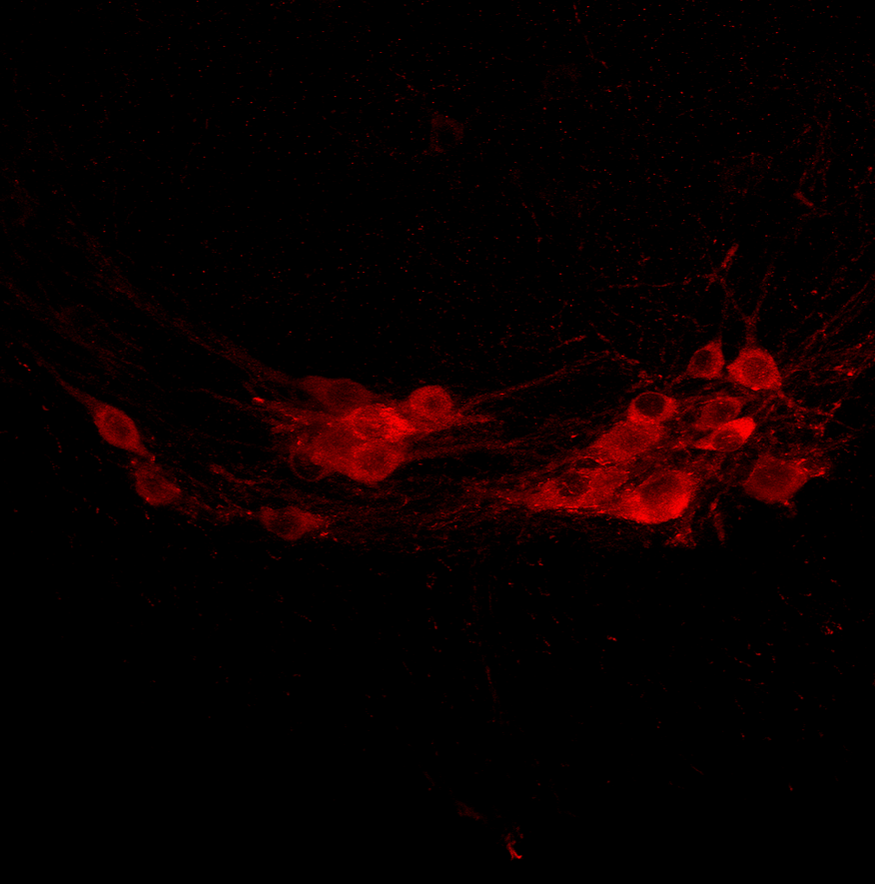

Supplement: Supplementary file 8 — Source data Fig. 5 [file 44321_2025_303_MOESM8_ESM.zip › Figure 5/5G/5G_L2_SMA+SMNC3(P0).tif]

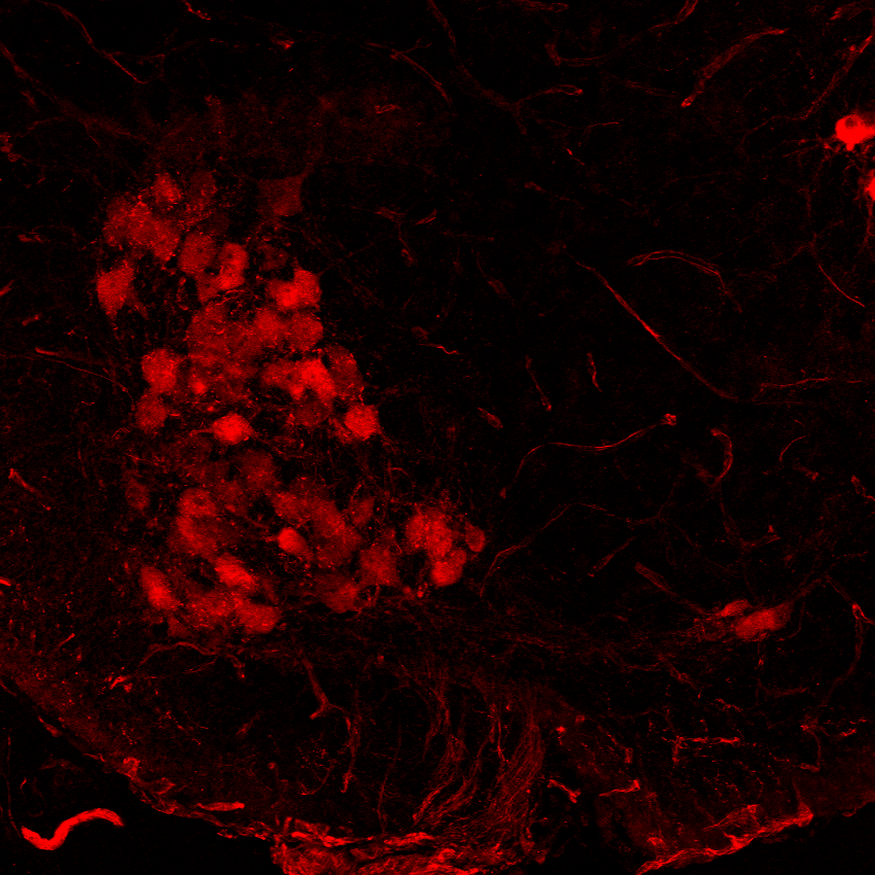

Supplement: Supplementary file 8 — Source data Fig. 5 [file 44321_2025_303_MOESM8_ESM.zip › Figure 5/5G/5G_L5_SMA+vehicle.tif]

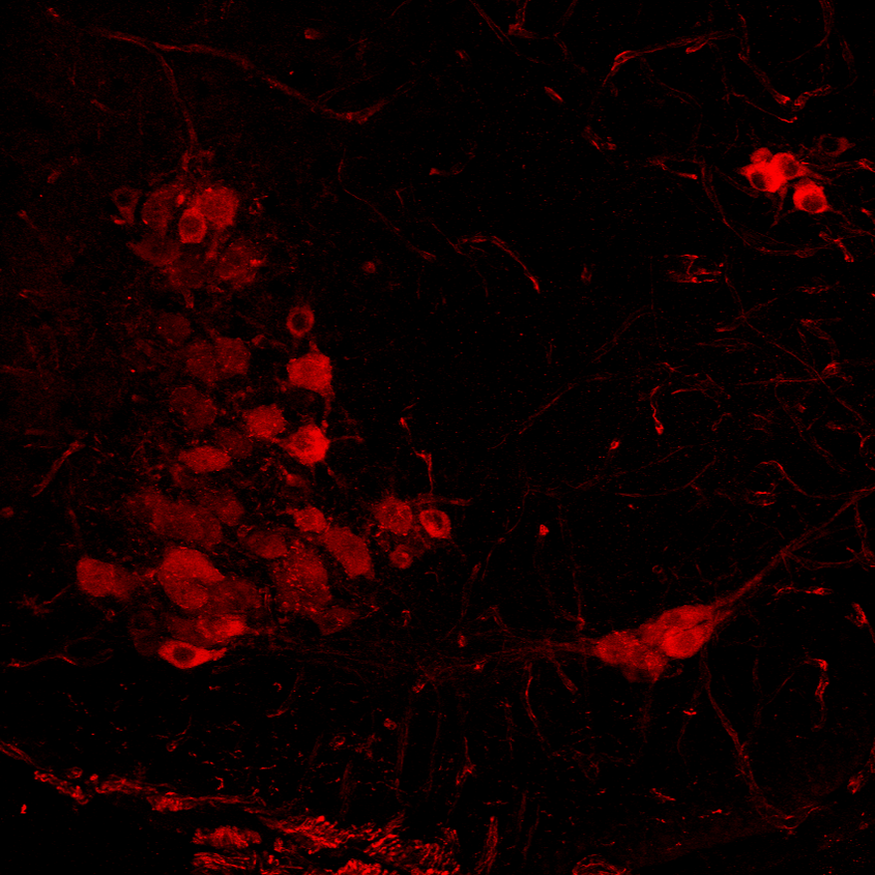

Supplement: Supplementary file 8 — Source data Fig. 5 [file 44321_2025_303_MOESM8_ESM.zip › Figure 5/5G/5G_L5_SMA+SMNC3(P0).tif]

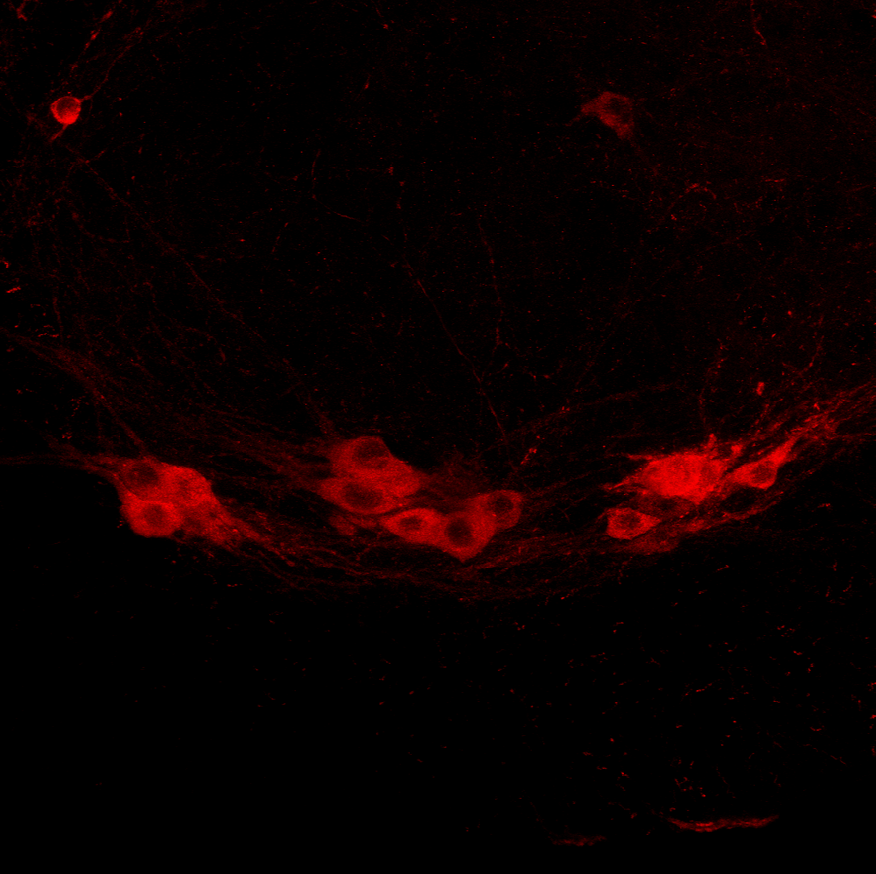

Supplement: Supplementary file 8 — Source data Fig. 5 [file 44321_2025_303_MOESM8_ESM.zip › Figure 5/5G/5G_L2_SMA+vehicle.tif]

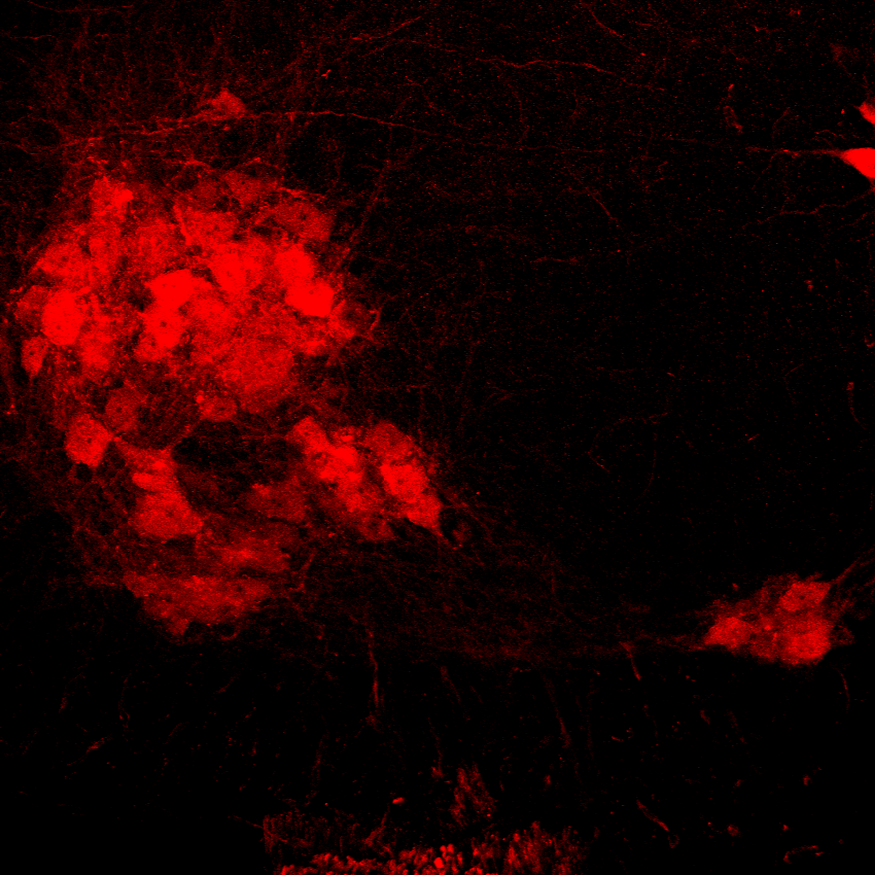

Supplement: Supplementary file 8 — Source data Fig. 5 [file 44321_2025_303_MOESM8_ESM.zip › Figure 5/5G/5G_L5_WT.tif]

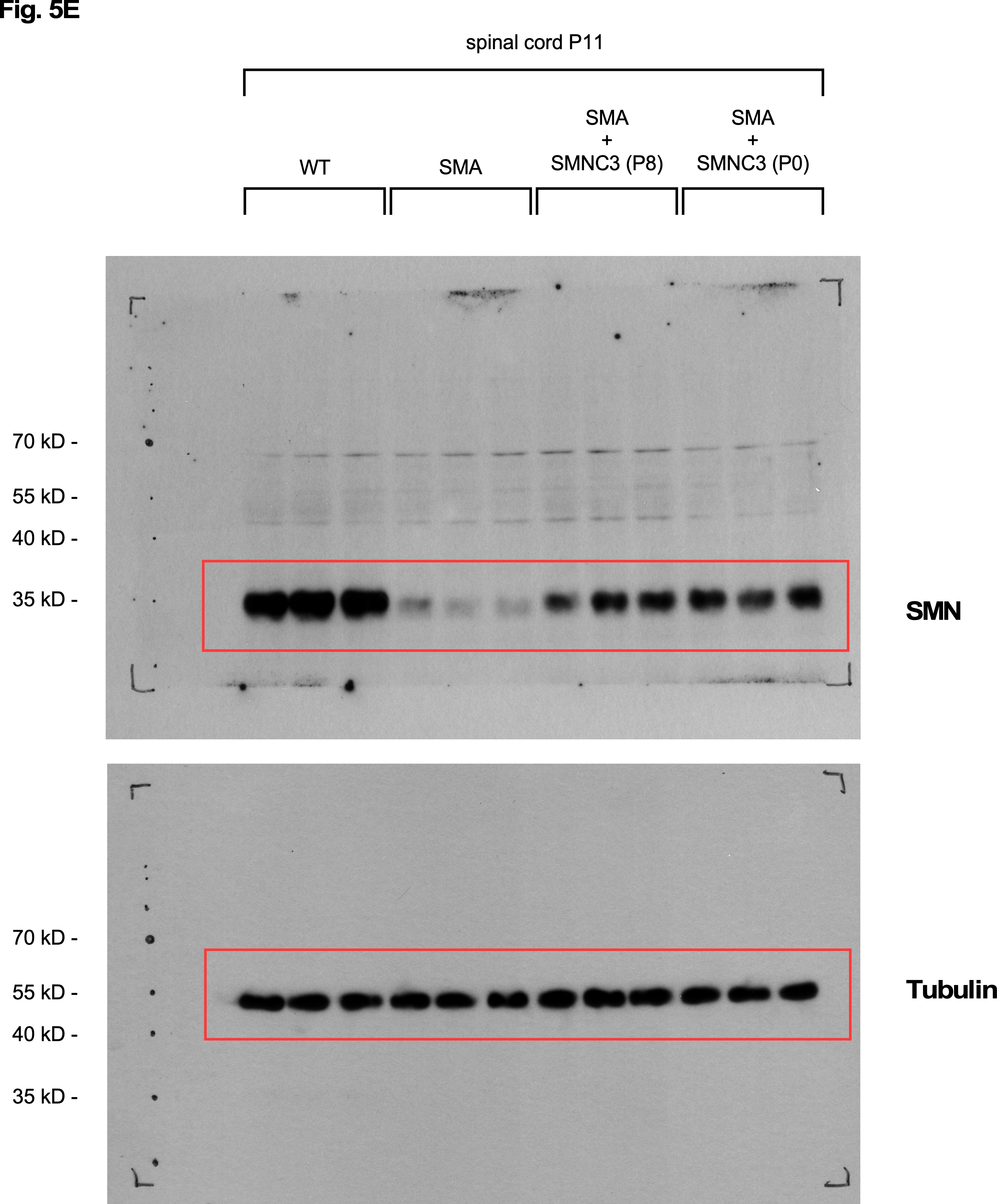

Supplement: Supplementary file 8 — Source data Fig. 5 [file 44321_2025_303_MOESM8_ESM.zip › Figure 5/5E/5E_WB.jpg]

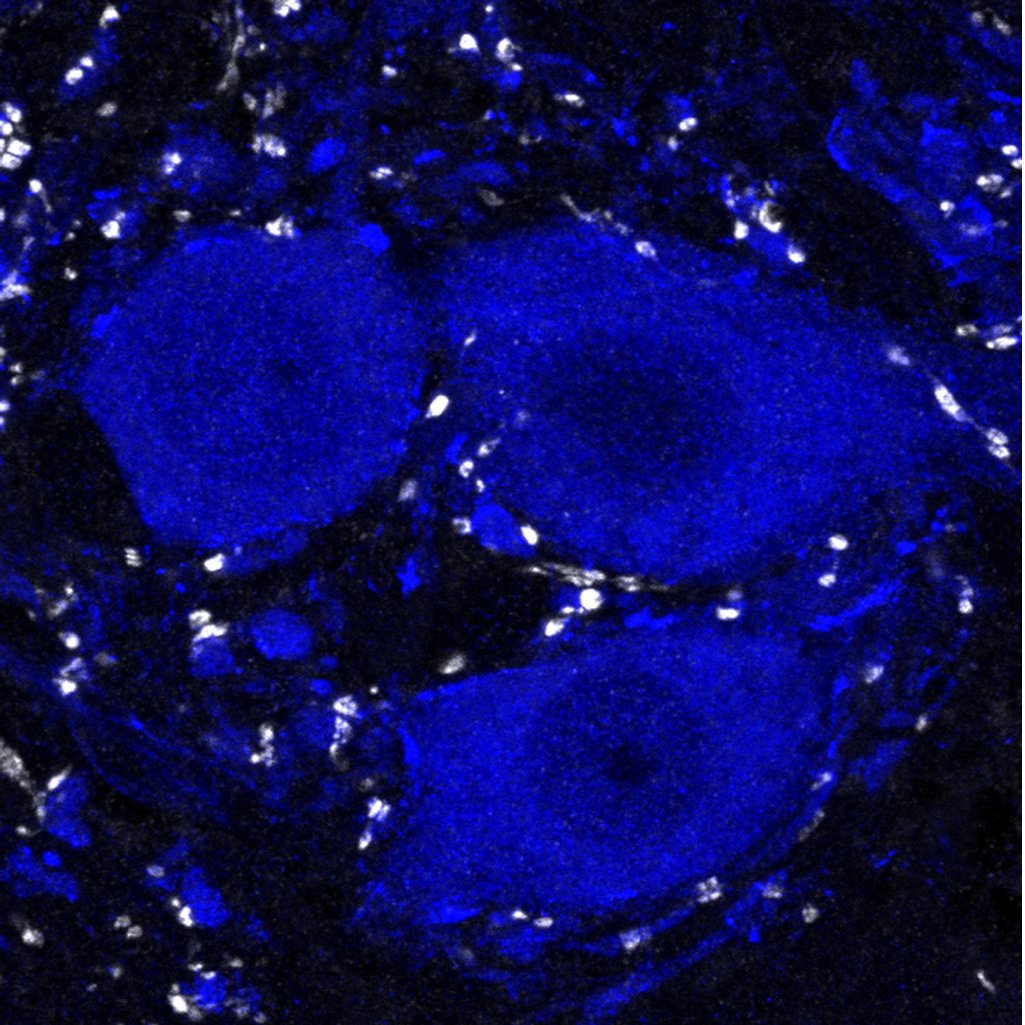

Supplement: Supplementary file 10 — Source data Fig. 7 [file 44321_2025_303_MOESM10_ESM.zip › Figure 7/7C/7C_SMA+SMNC3(P0).tif]

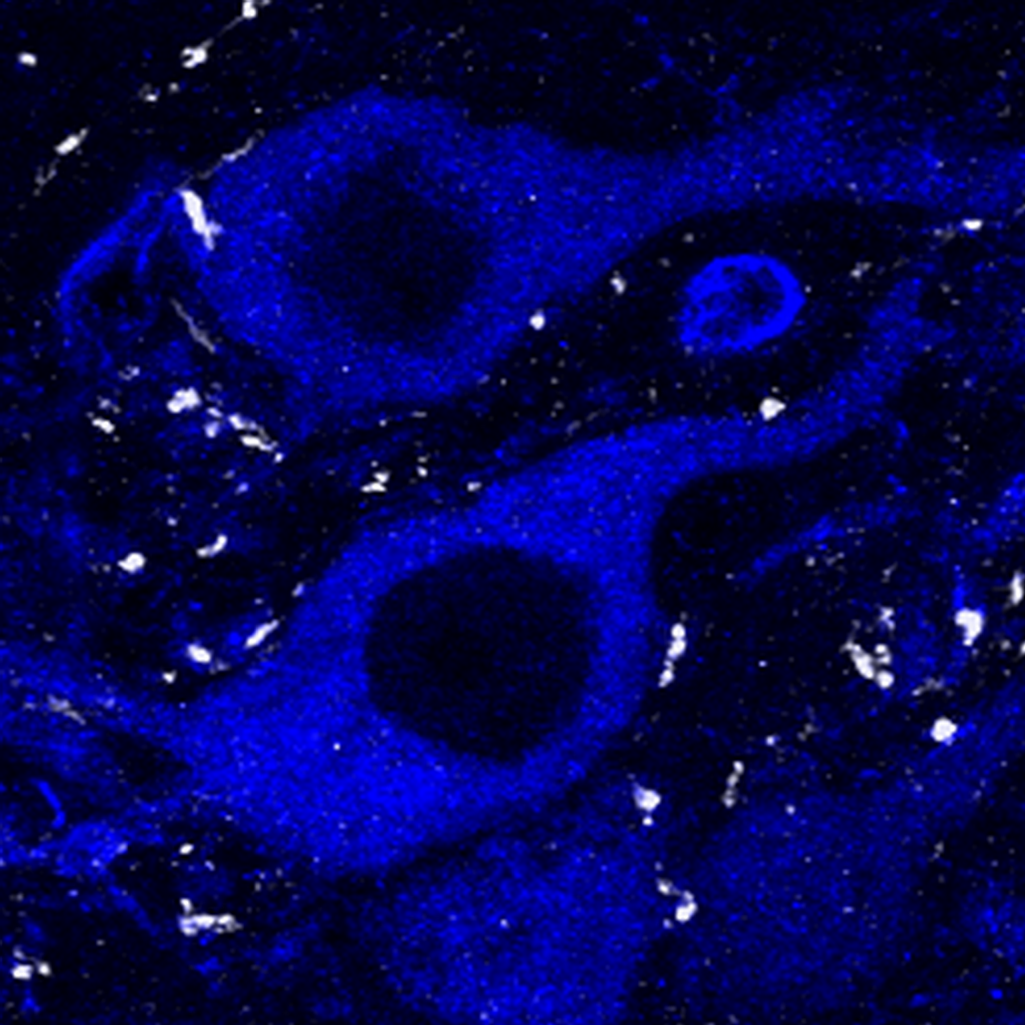

Supplement: Supplementary file 10 — Source data Fig. 7 [file 44321_2025_303_MOESM10_ESM.zip › Figure 7/7C/7C_SMA+MW150(P0).tif]

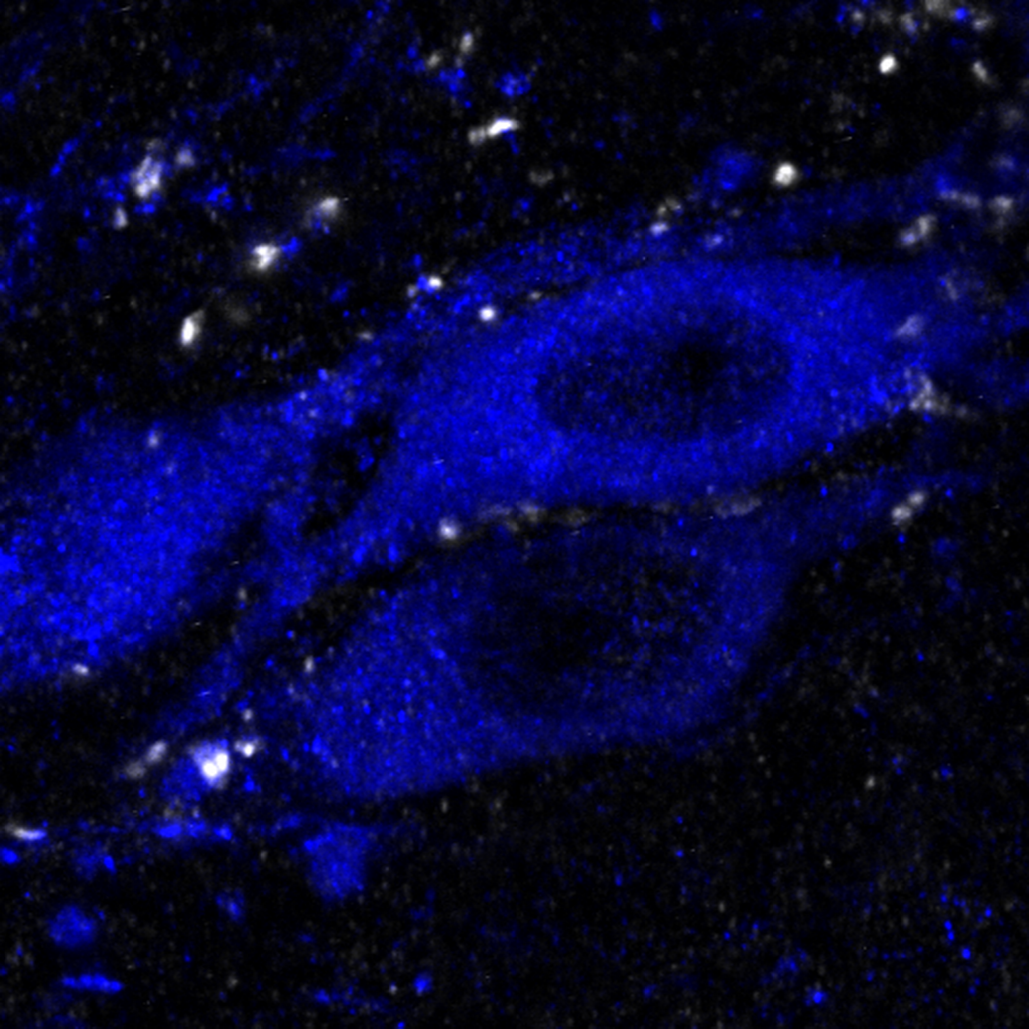

Supplement: Supplementary file 10 — Source data Fig. 7 [file 44321_2025_303_MOESM10_ESM.zip › Figure 7/7C/7C_SMA+SMNC3(P8)+MW150(P0).tif]

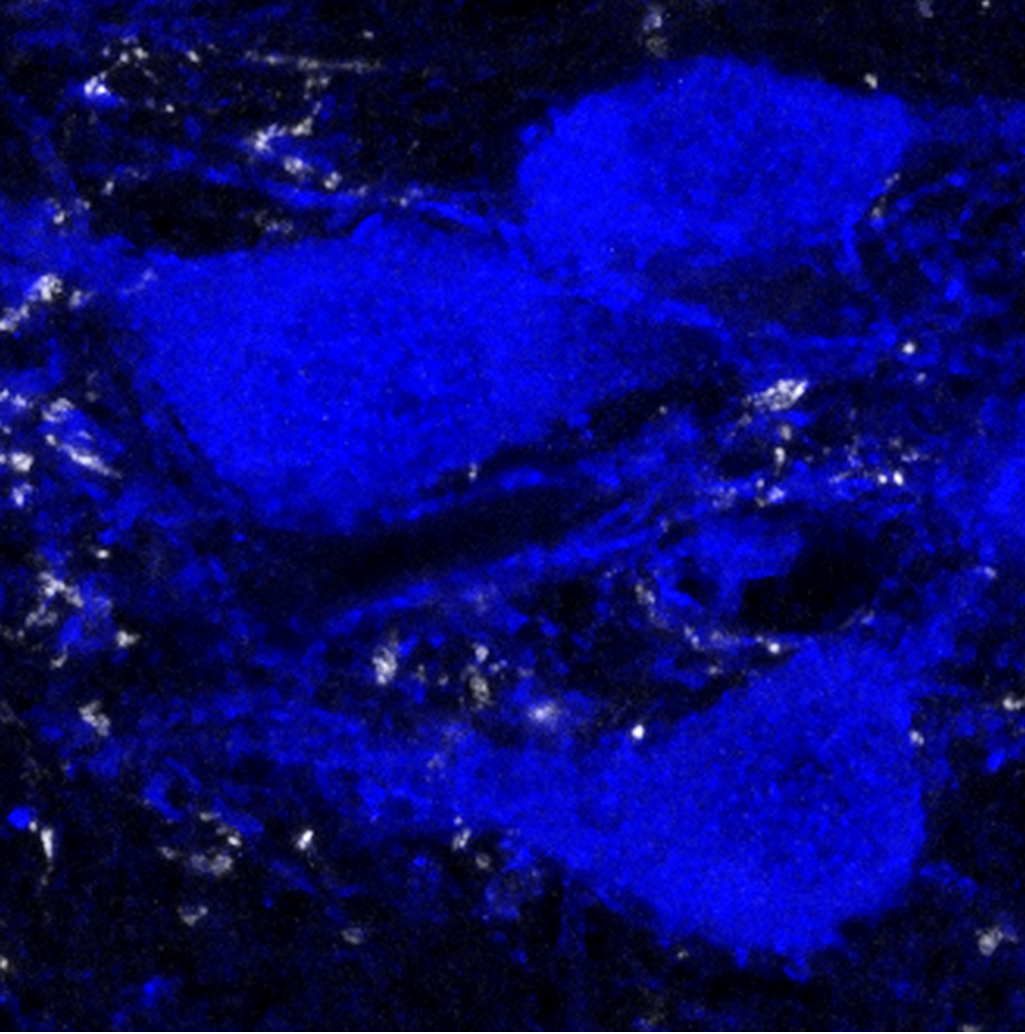

Supplement: Supplementary file 10 — Source data Fig. 7 [file 44321_2025_303_MOESM10_ESM.zip › Figure 7/7C/7C_SMA+SMNC3(P8).tif]

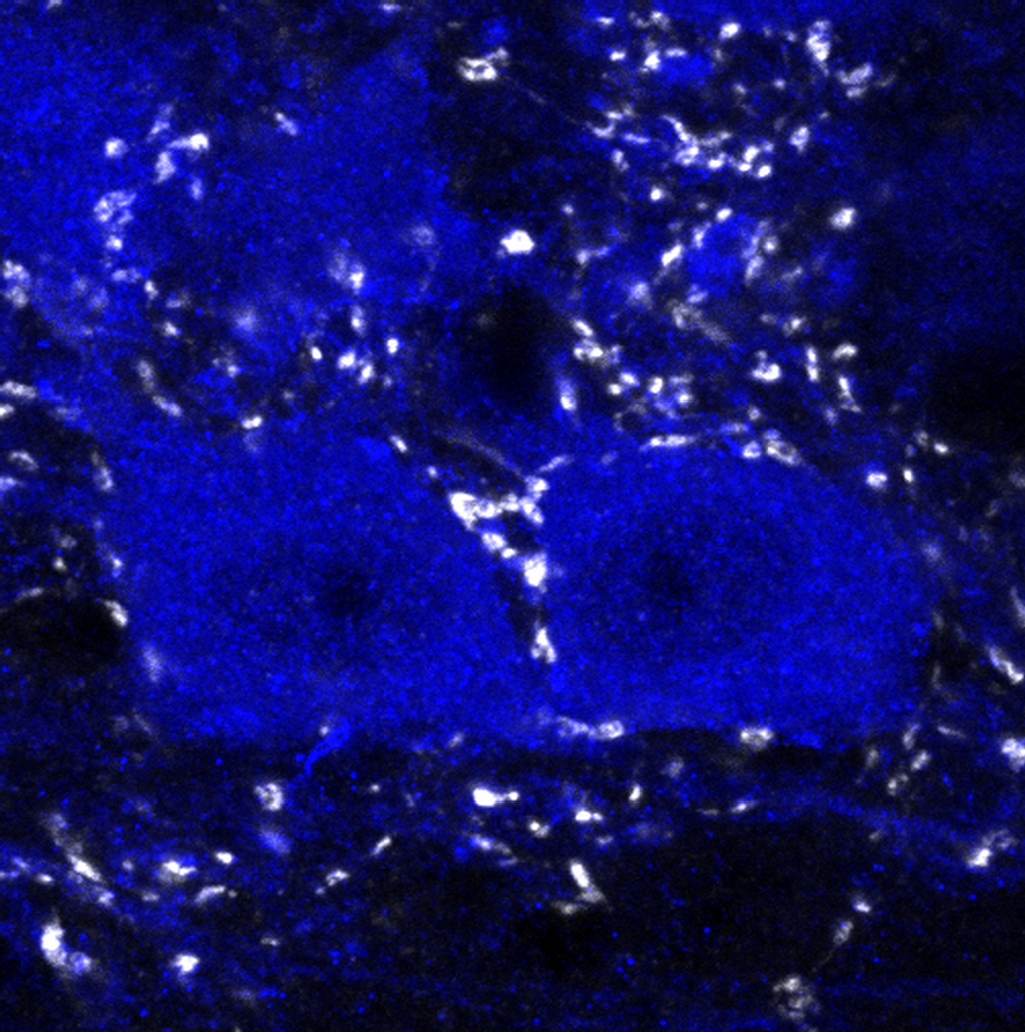

Supplement: Supplementary file 10 — Source data Fig. 7 [file 44321_2025_303_MOESM10_ESM.zip › Figure 7/7C/7C_WT.tif]

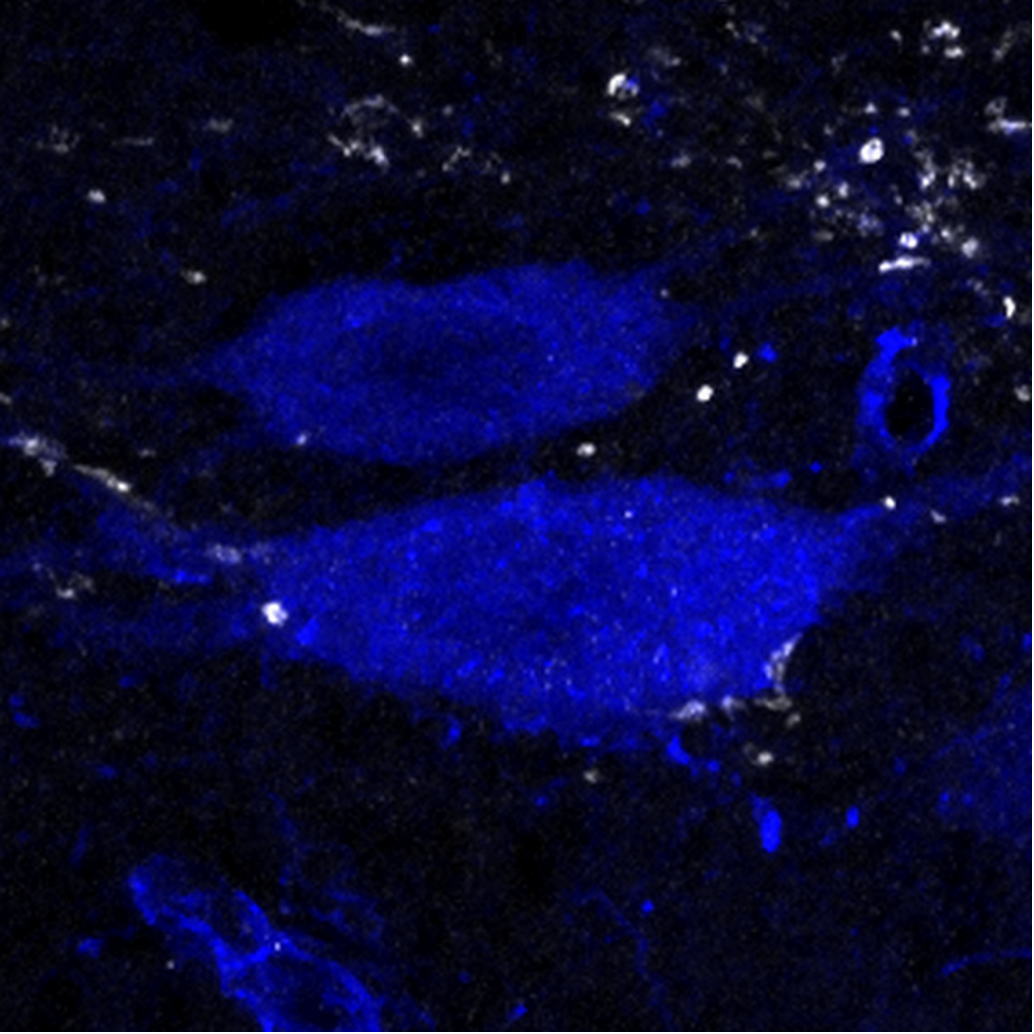

Supplement: Supplementary file 10 — Source data Fig. 7 [file 44321_2025_303_MOESM10_ESM.zip › Figure 7/7C/7C_SMA+vehicle.tif]

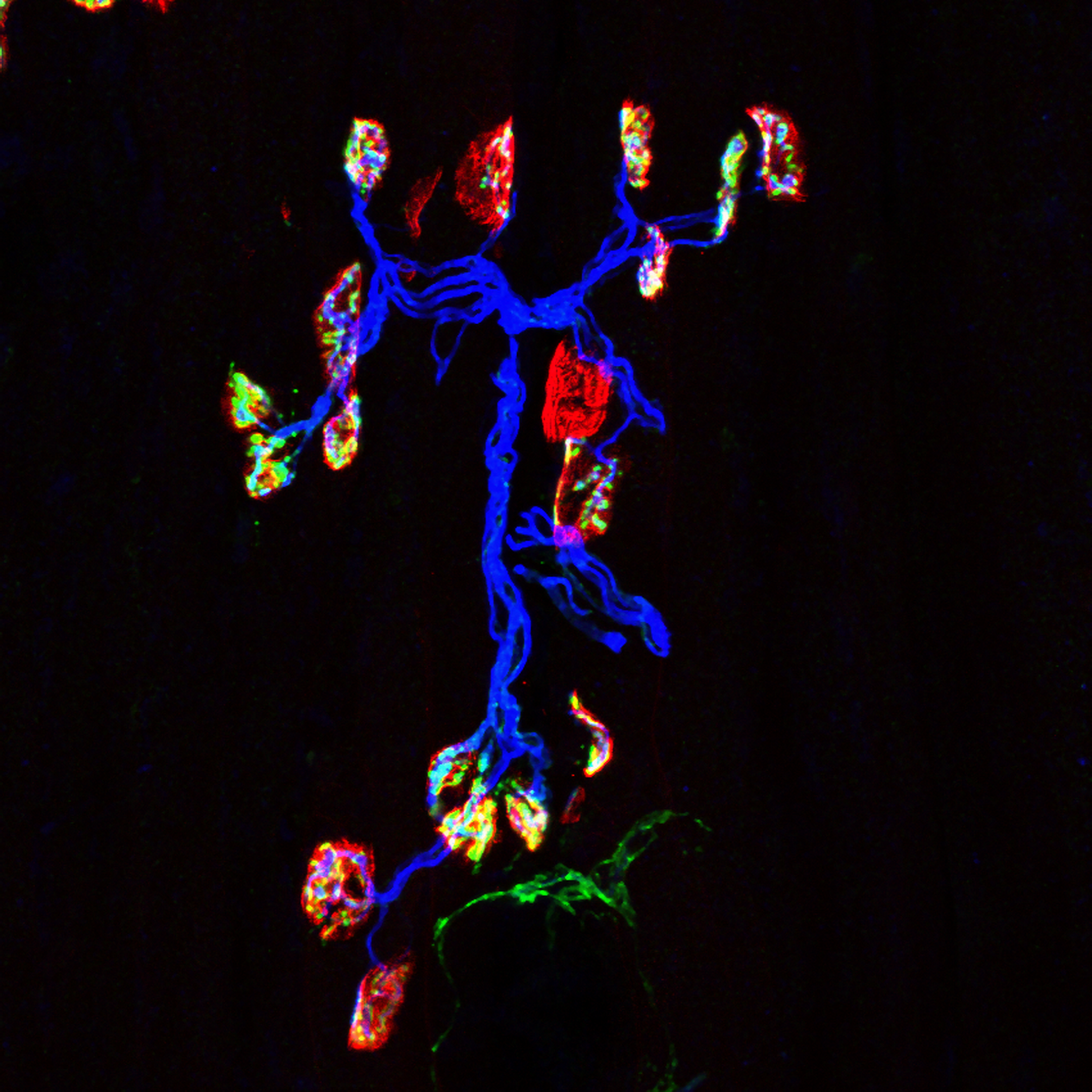

Supplement: Supplementary file 10 — Source data Fig. 7 [file 44321_2025_303_MOESM10_ESM.zip › Figure 7/7A/7A_SMA+MW150(P0):SMNC3(P8).tif]

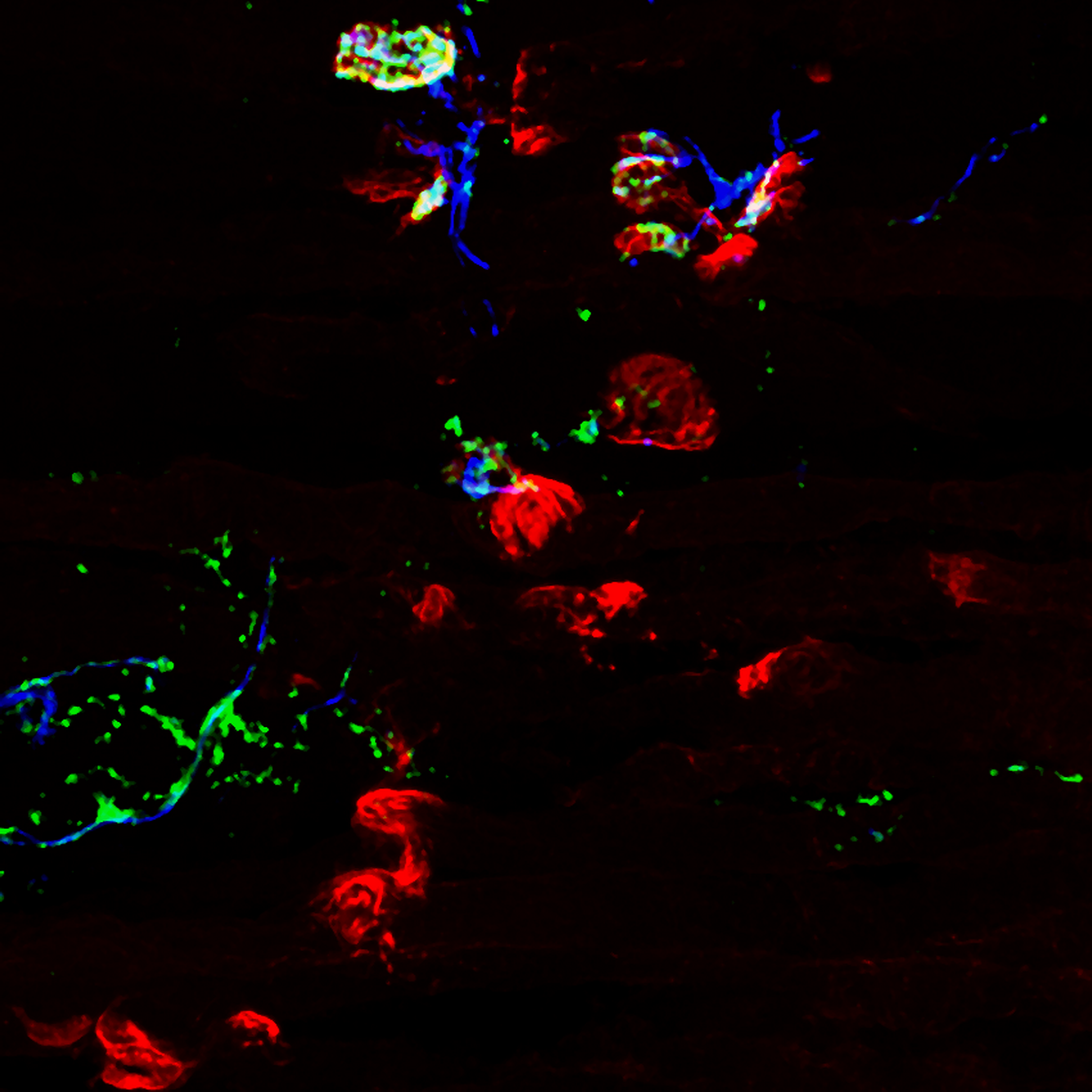

Supplement: Supplementary file 10 — Source data Fig. 7 [file 44321_2025_303_MOESM10_ESM.zip › Figure 7/7A/7A_SMA+vehicle.tif]

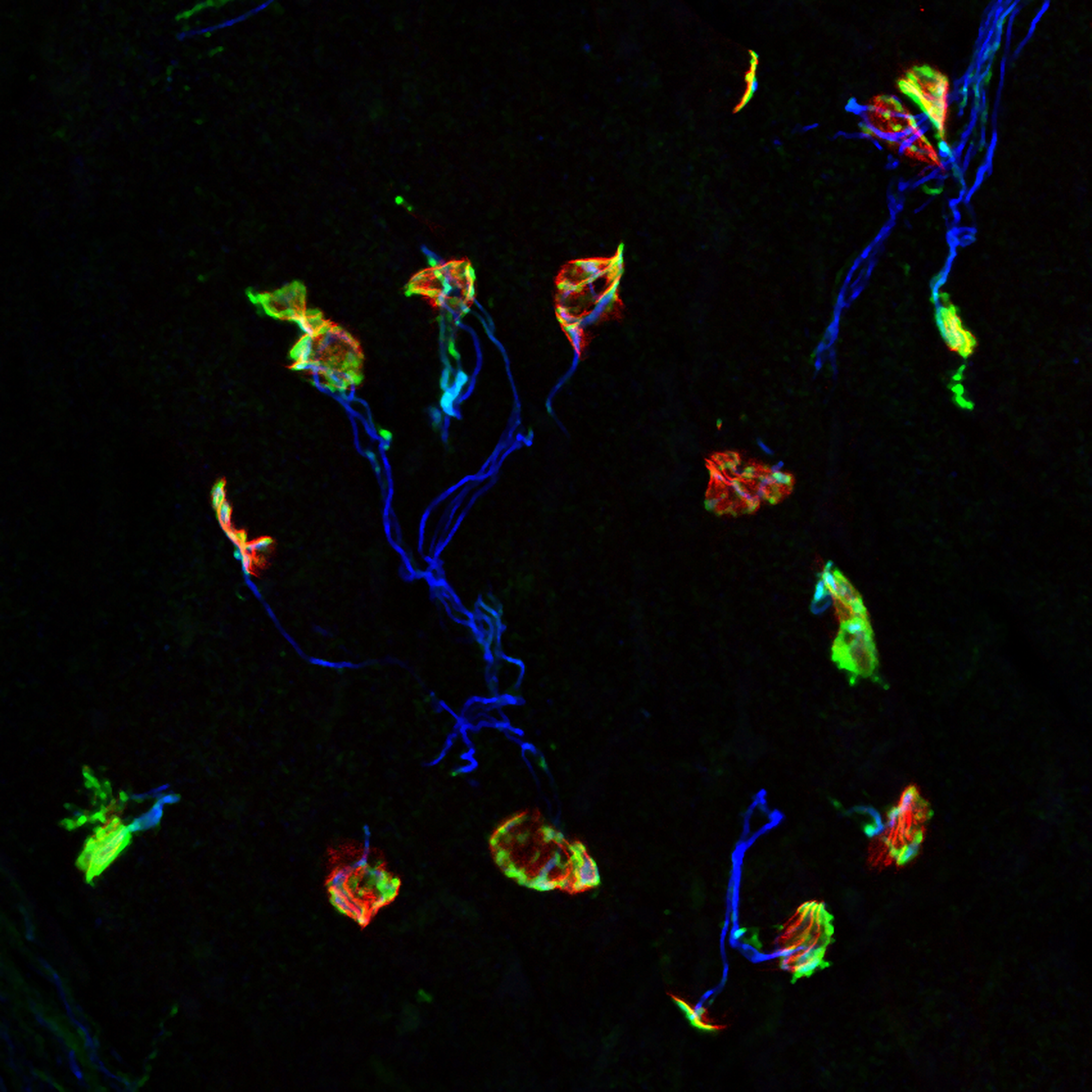

Supplement: Supplementary file 10 — Source data Fig. 7 [file 44321_2025_303_MOESM10_ESM.zip › Figure 7/7A/7A_SMA+SMNC3(P0).tif]

Figure 7A

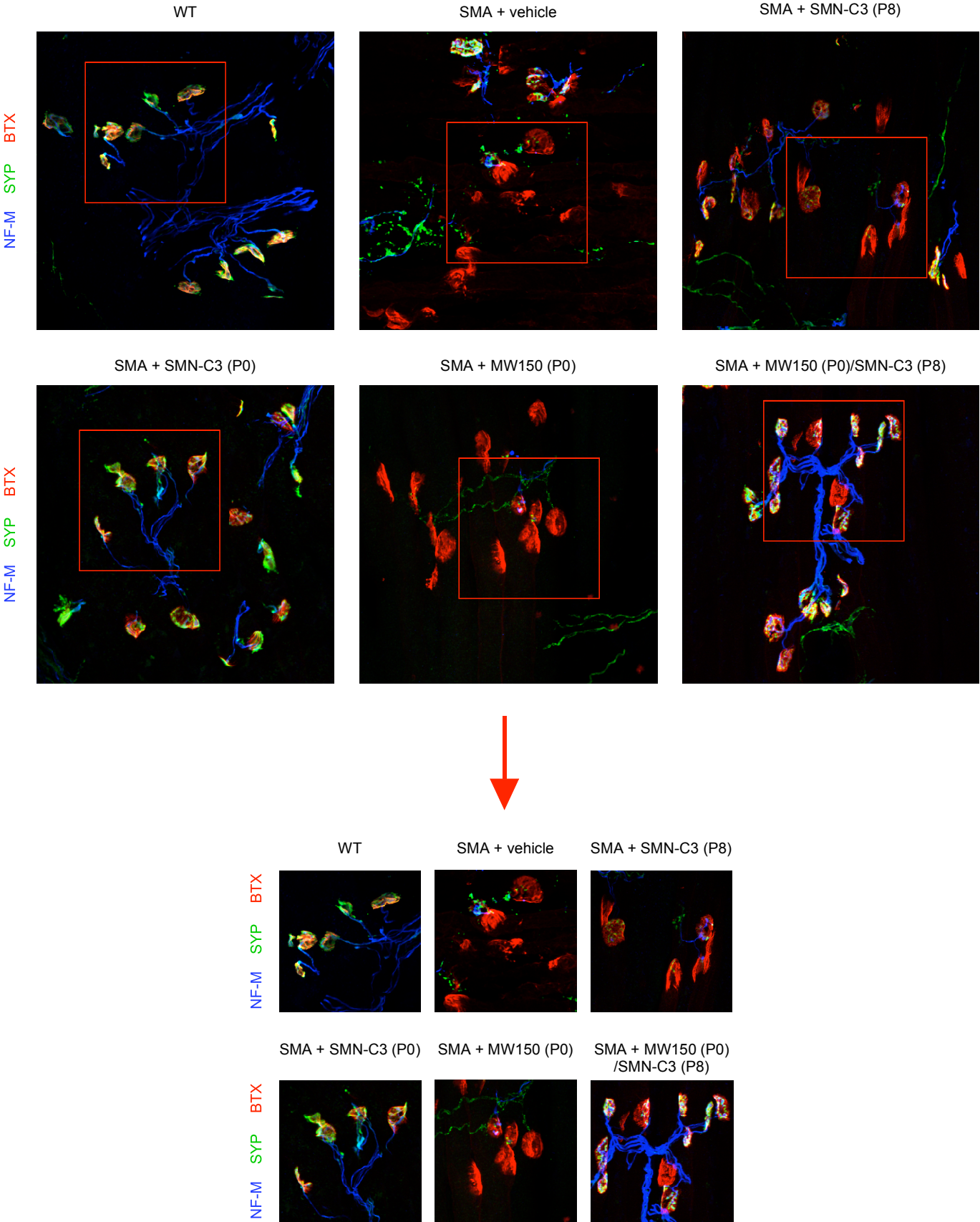

Supplement: Supplementary file 10 — Source data Fig. 7 [file 44321_2025_303_MOESM10_ESM.zip › Figure 7/7A/Figure 7A README.pdf]

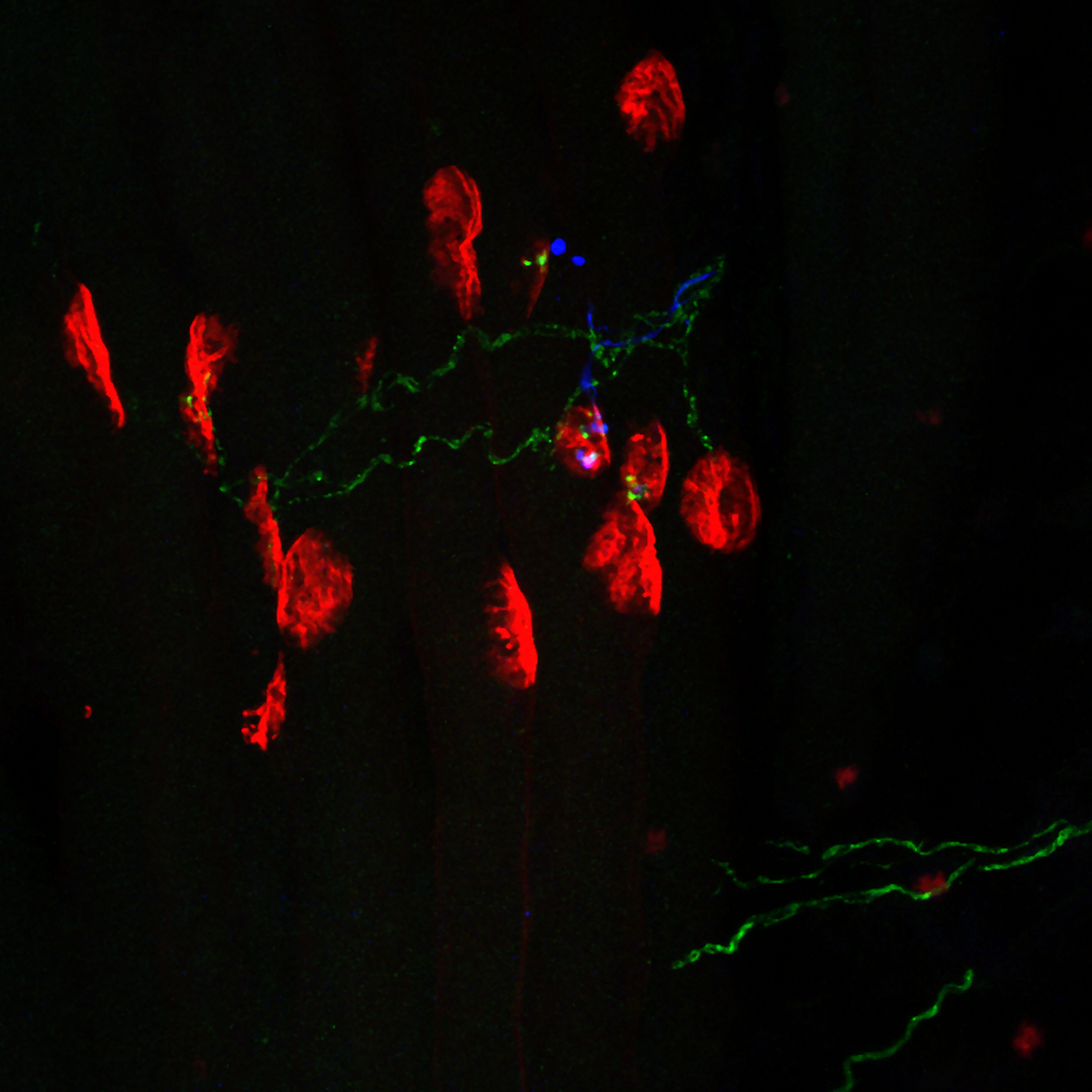

Supplement: Supplementary file 10 — Source data Fig. 7 [file 44321_2025_303_MOESM10_ESM.zip › Figure 7/7A/7A_SMA+MW150(P0).tif]

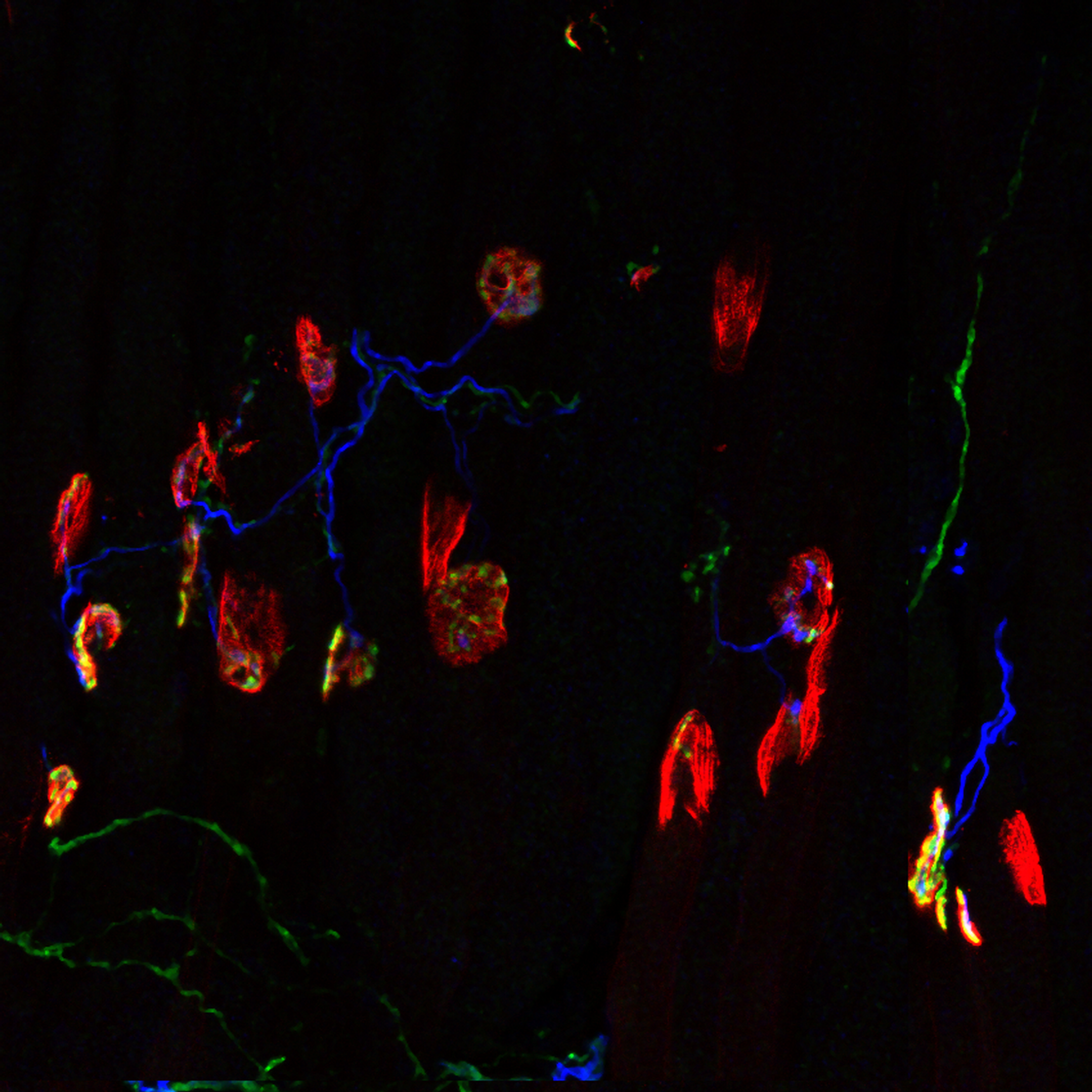

Supplement: Supplementary file 10 — Source data Fig. 7 [file 44321_2025_303_MOESM10_ESM.zip › Figure 7/7A/7A_SMA+SMNC3(P8).tif]

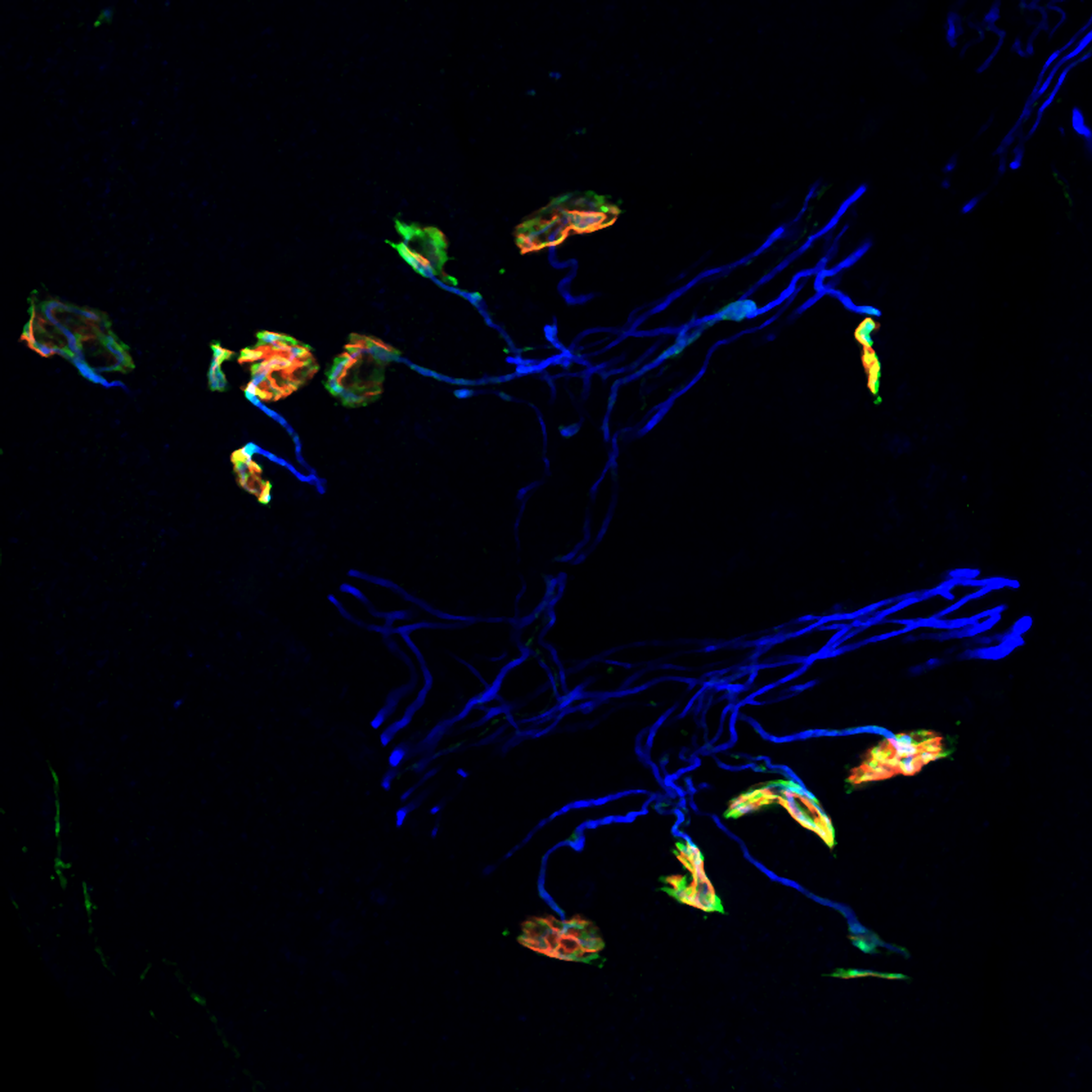

Supplement: Supplementary file 10 — Source data Fig. 7 [file 44321_2025_303_MOESM10_ESM.zip › Figure 7/7A/7A_WT.tif]

Figure 8A

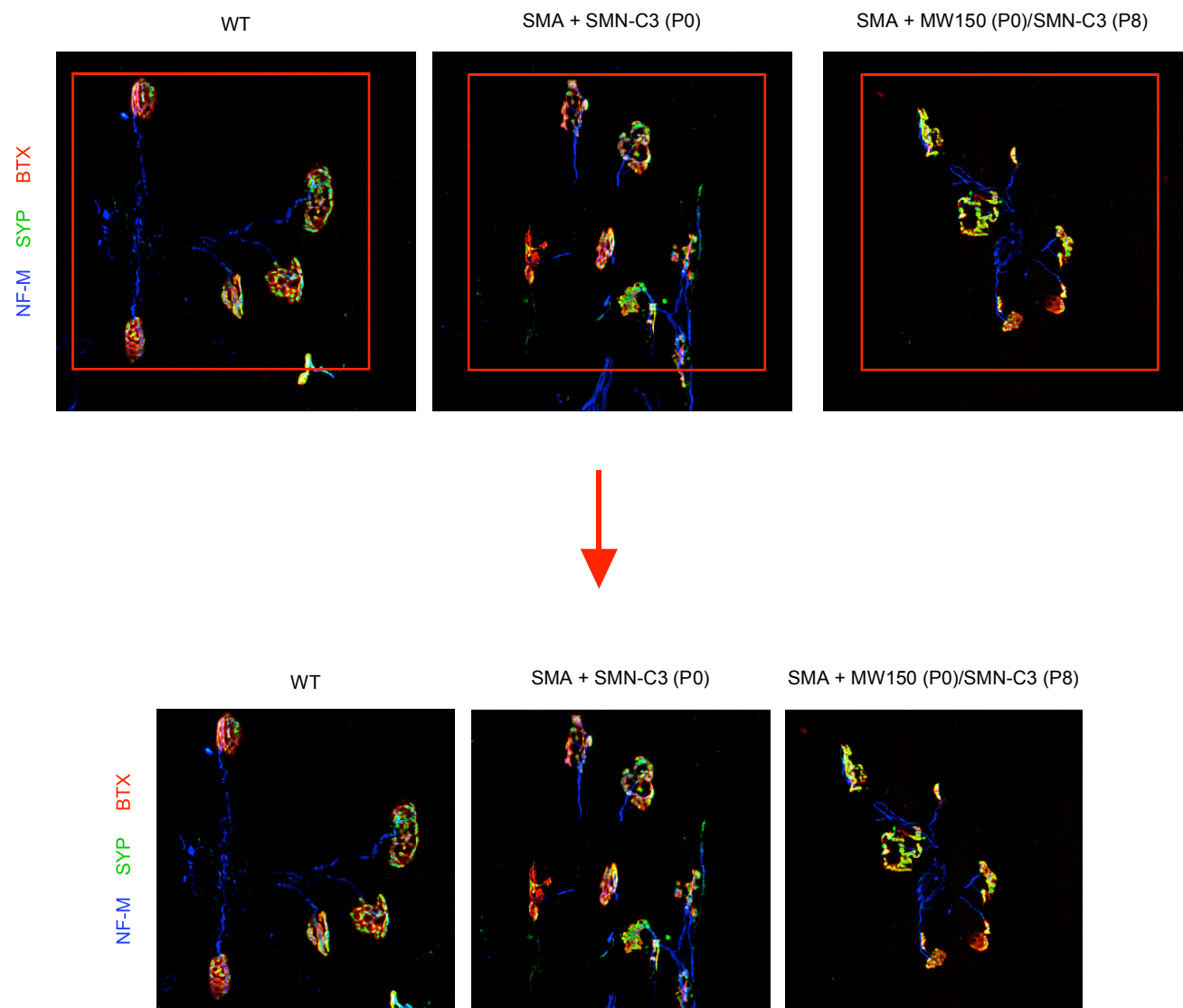

Supplement: Supplementary file 11 — Source data Fig. 8 [file 44321_2025_303_MOESM11_ESM.zip › Figure 8/8A/Figure 8A README.pdf]

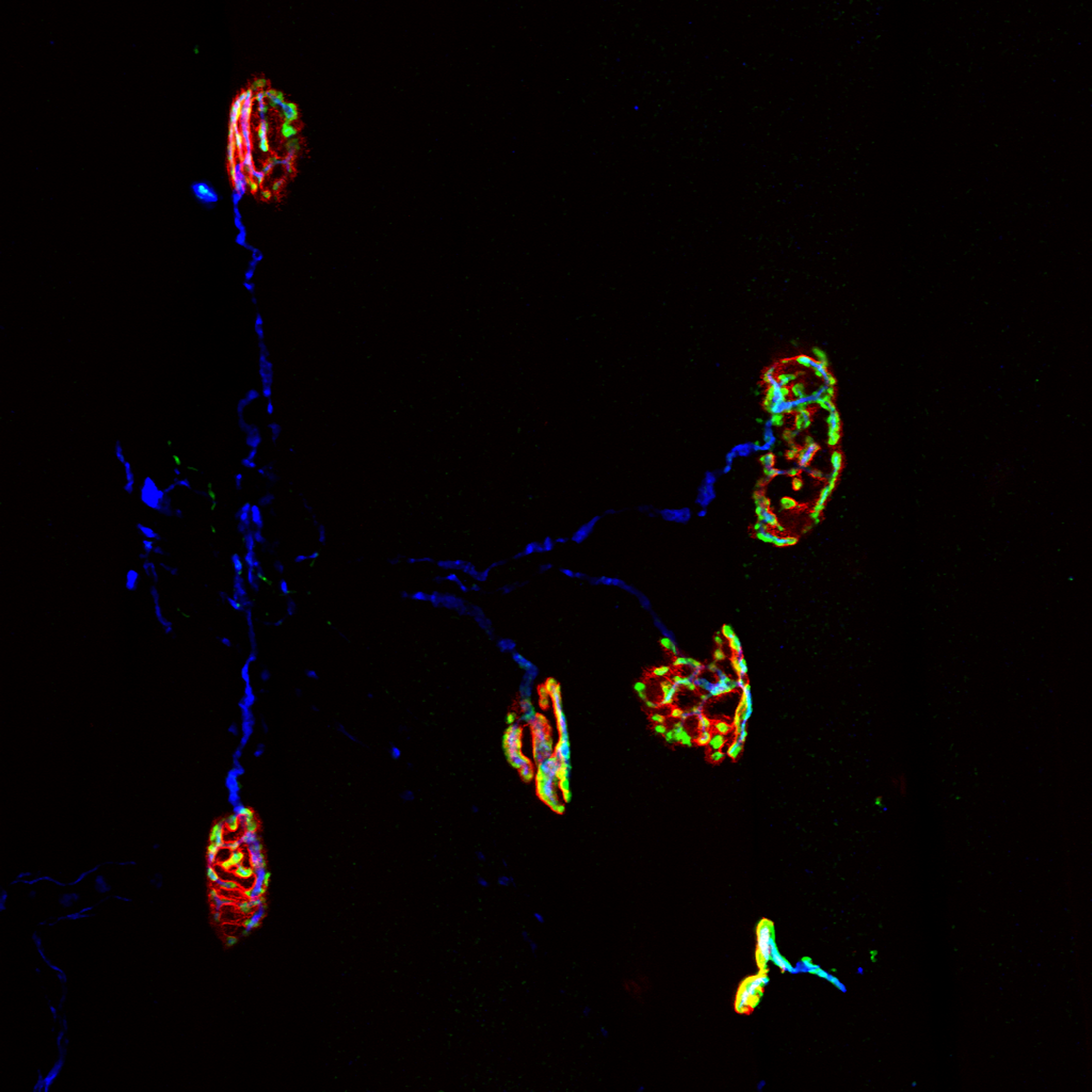

Supplement: Supplementary file 11 — Source data Fig. 8 [file 44321_2025_303_MOESM11_ESM.zip › Figure 8/8A/8A_WT.tif]

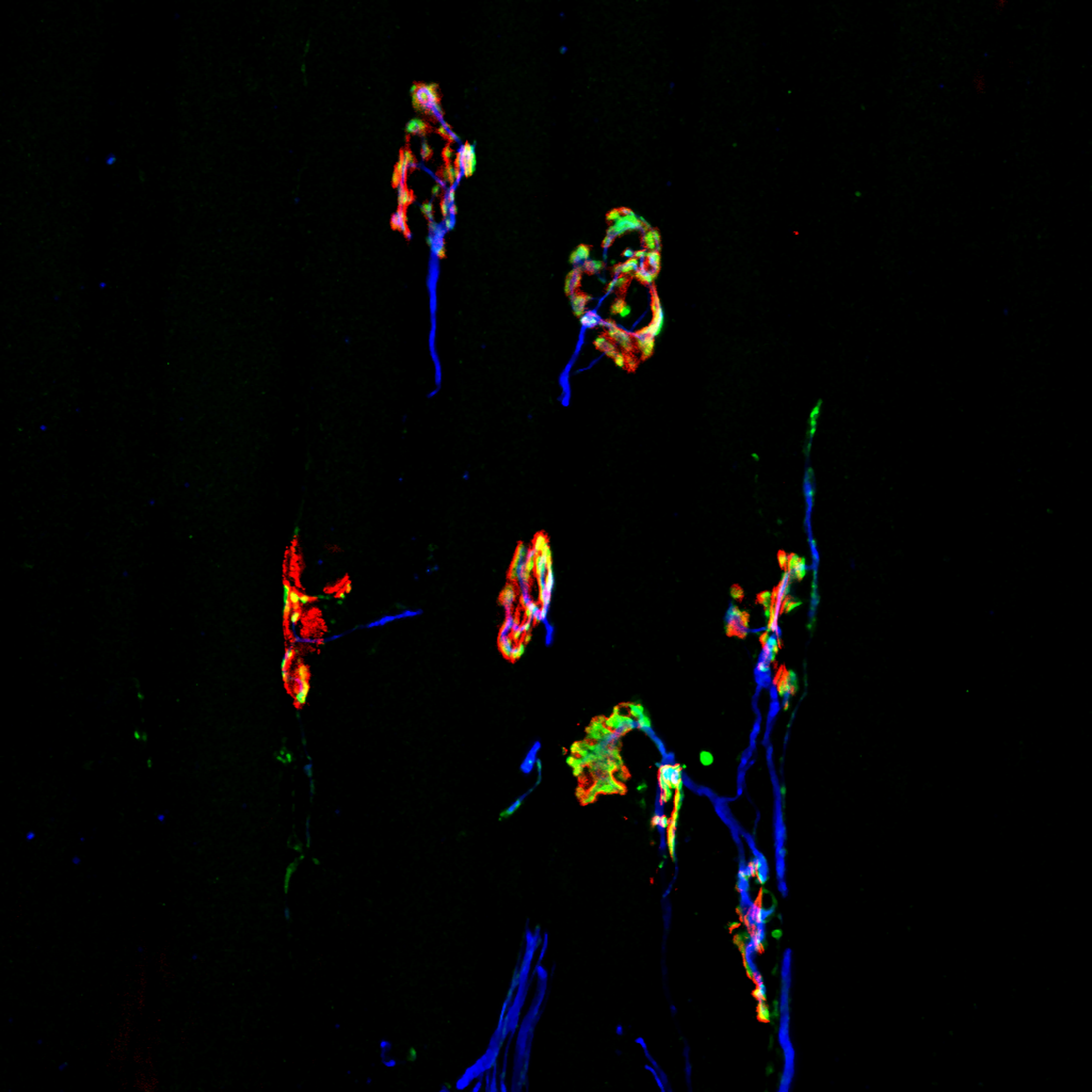

Supplement: Supplementary file 11 — Source data Fig. 8 [file 44321_2025_303_MOESM11_ESM.zip › Figure 8/8A/8A_SMA + SMN-C3 (P0).tif]

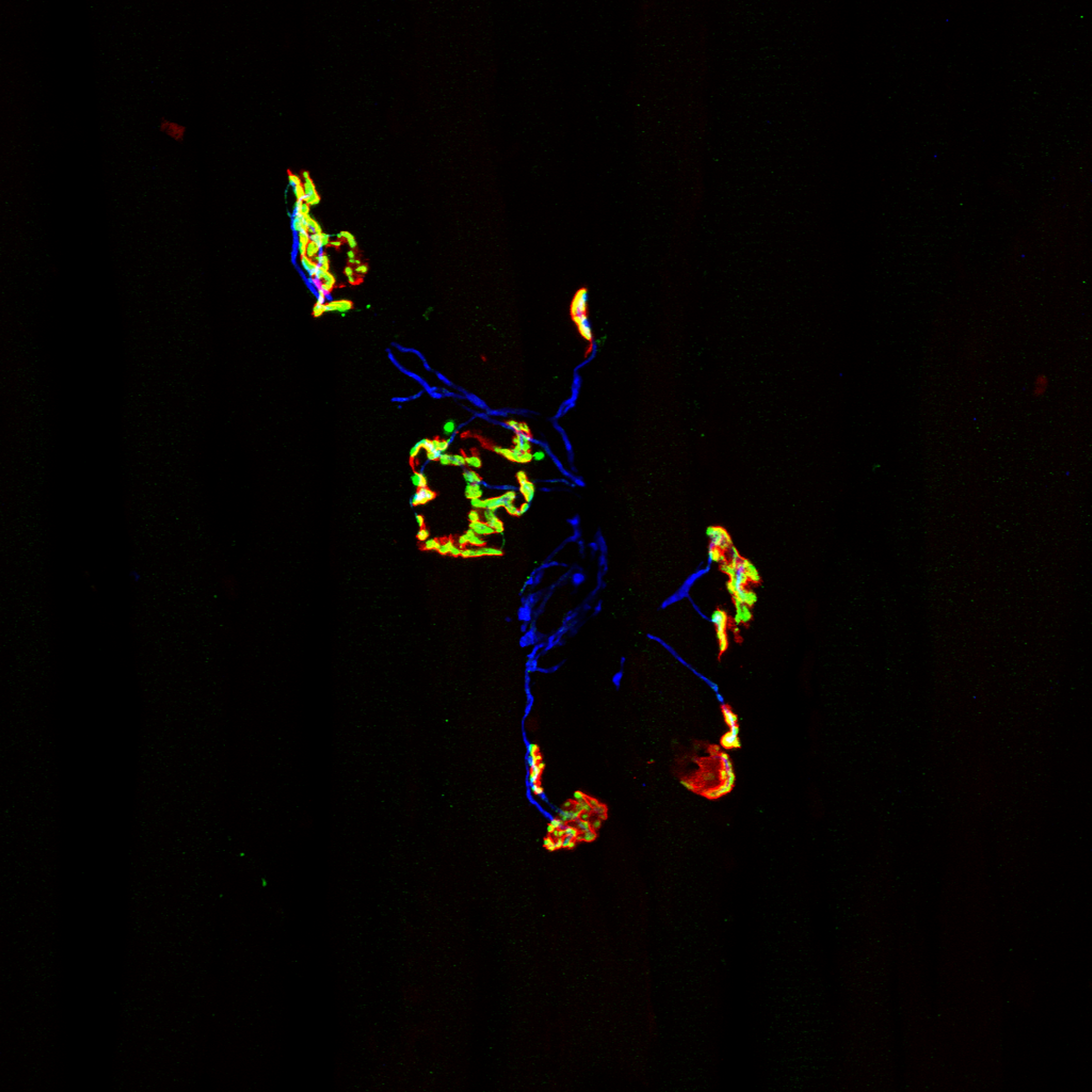

Supplement: Supplementary file 11 — Source data Fig. 8 [file 44321_2025_303_MOESM11_ESM.zip › Figure 8/8A/8A_SMA + MW150 (P0) : SMN-C3 (P8).tif]

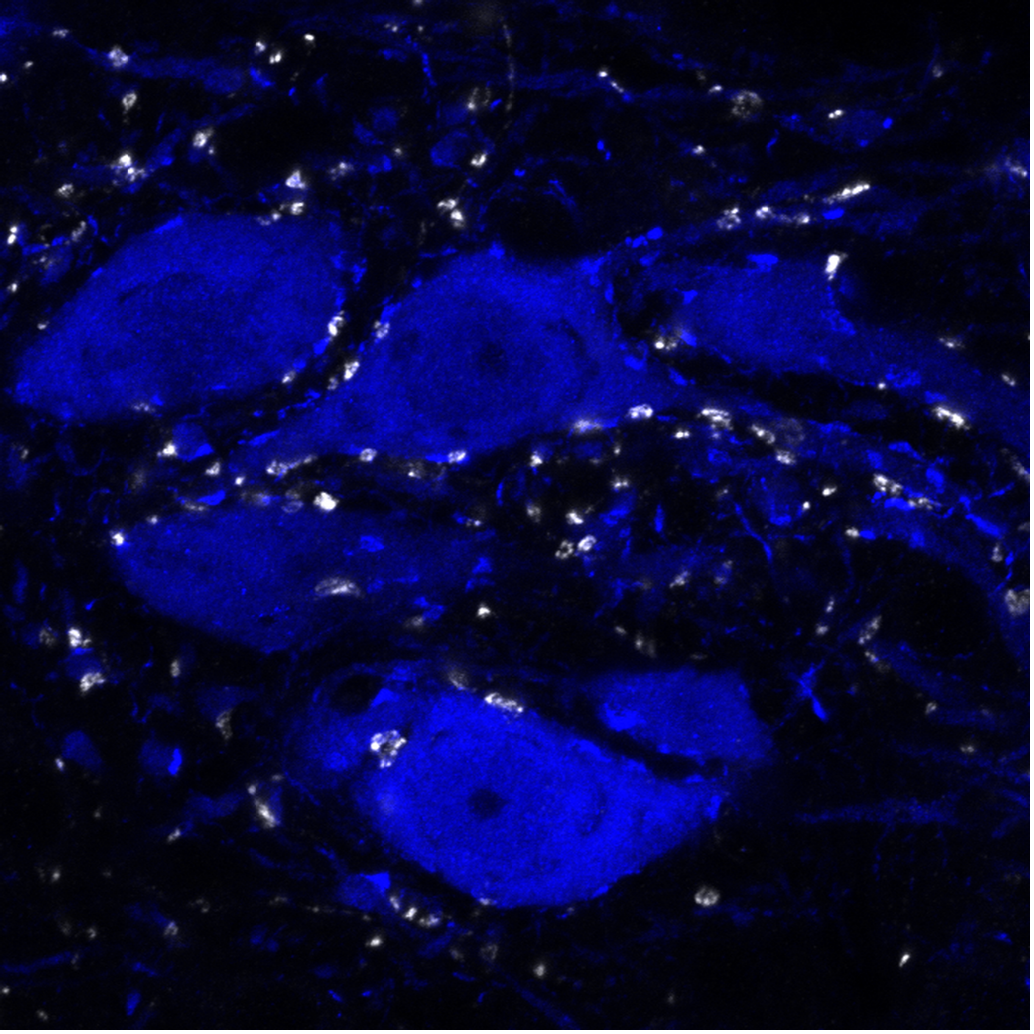

Supplement: Supplementary file 11 — Source data Fig. 8 [file 44321_2025_303_MOESM11_ESM.zip › Figure 8/8C/8C_WT.tif]

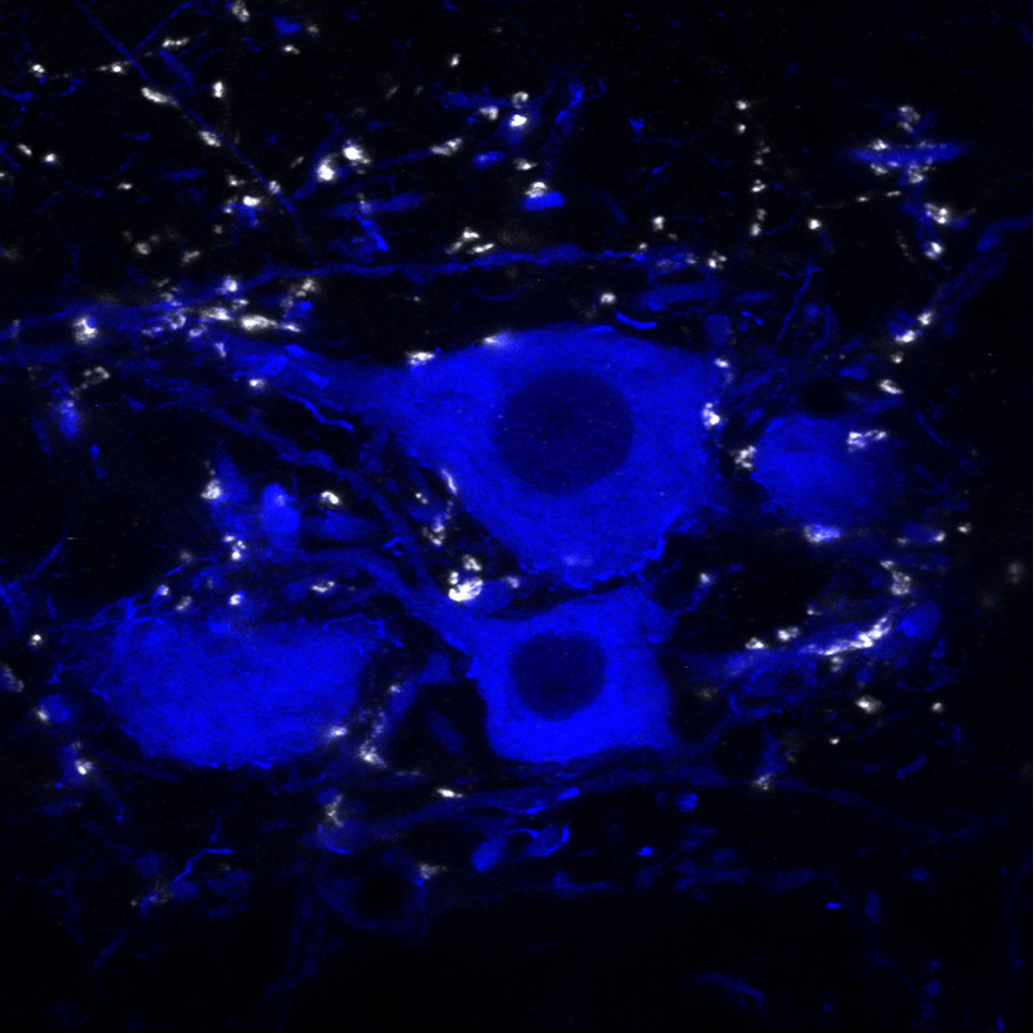

Supplement: Supplementary file 11 — Source data Fig. 8 [file 44321_2025_303_MOESM11_ESM.zip › Figure 8/8C/8C_SMA+SMNC3(P8)+MW150(P0).tif]

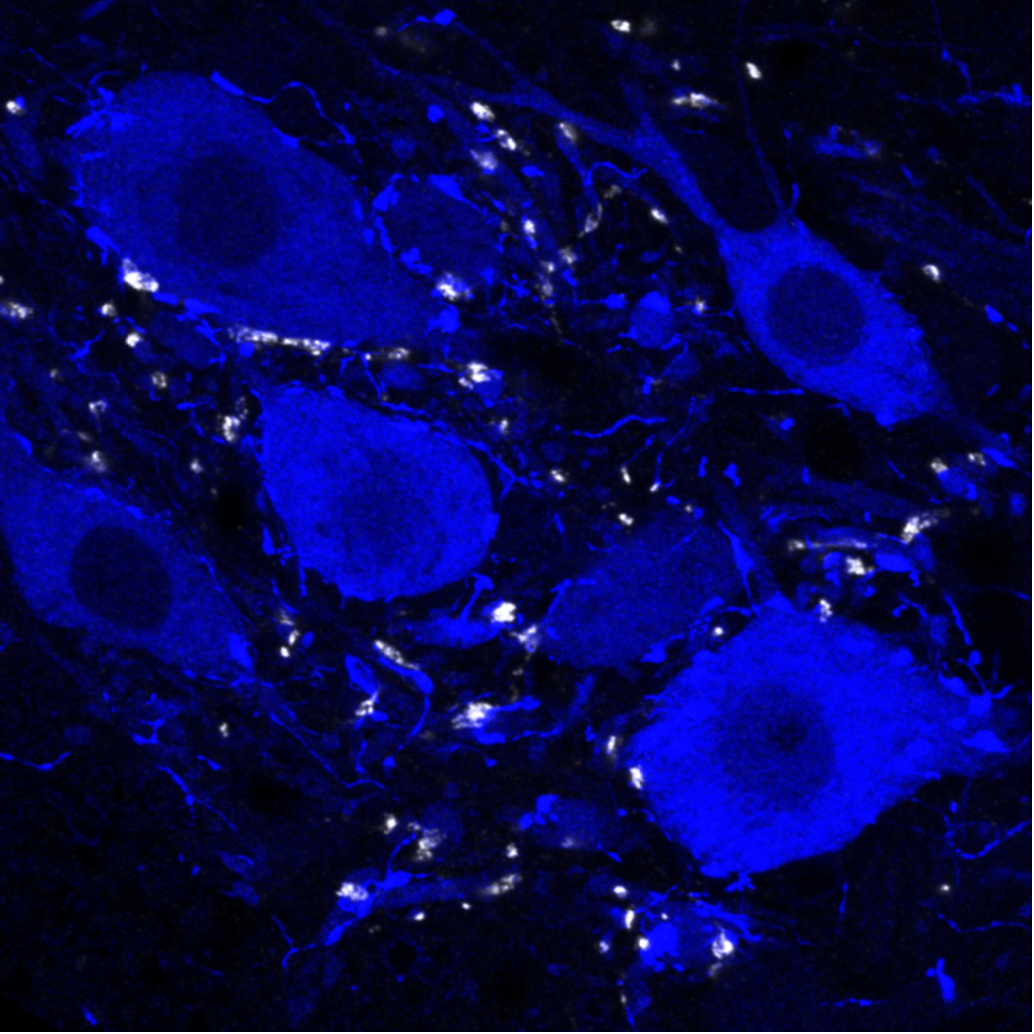

Supplement: Supplementary file 11 — Source data Fig. 8 [file 44321_2025_303_MOESM11_ESM.zip › Figure 8/8C/8C_SMA+SMNC3(P0).tif]

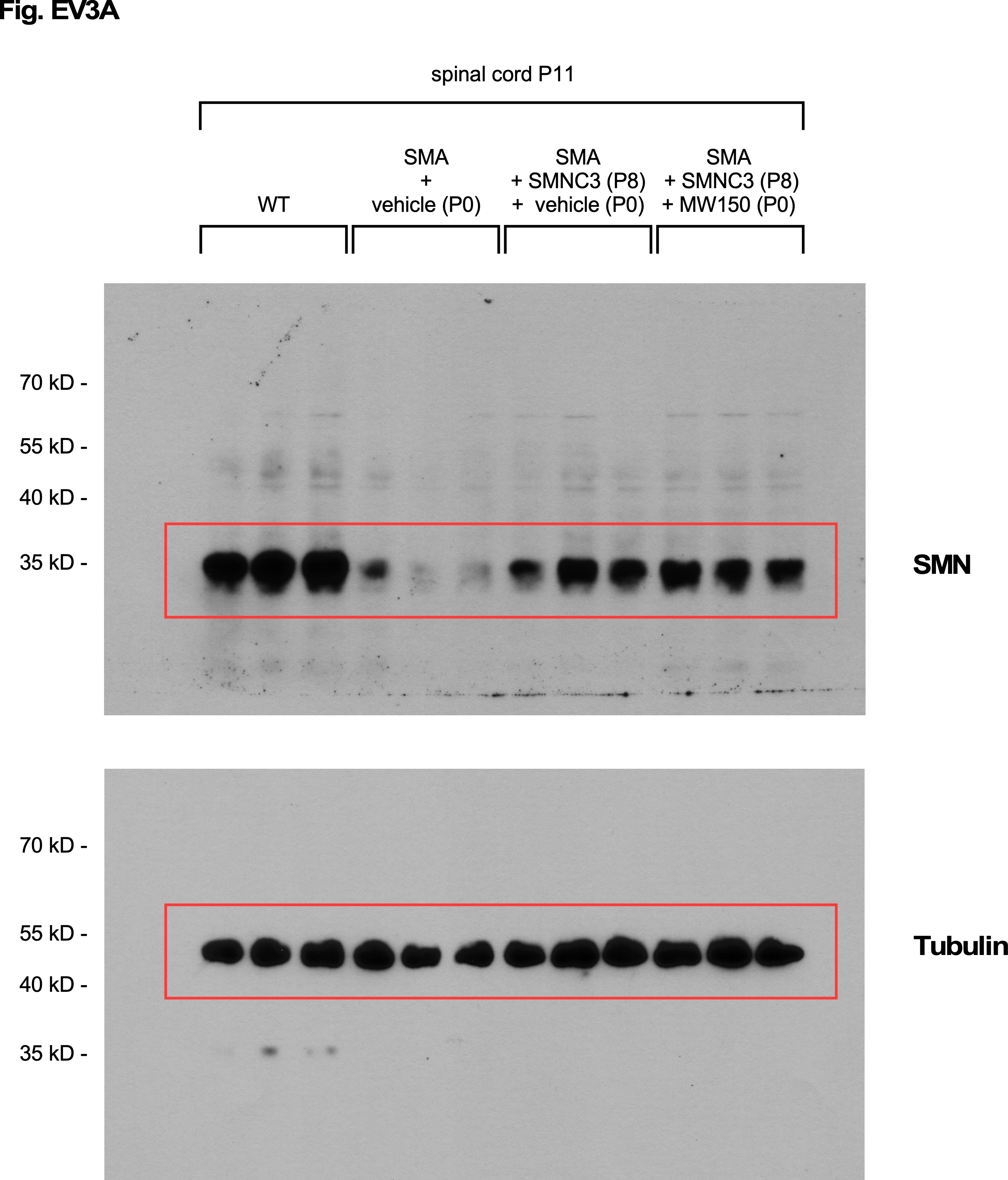

Supplement: Supplementary file 14 — Figure EV3 Source Data [file 44321_2025_303_MOESM14_ESM.zip › Figure EV3/EV3A/EV3A_WB.jpg]

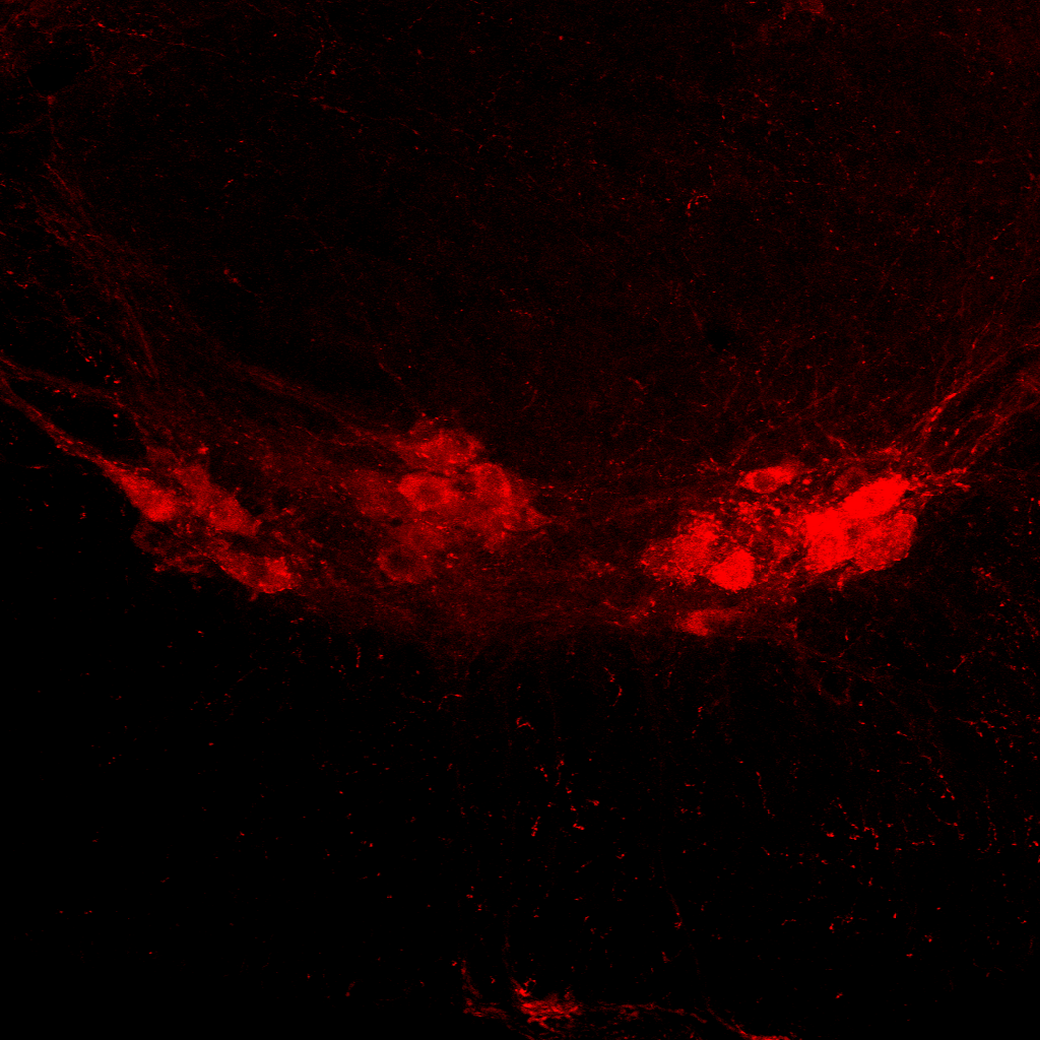

Supplement: Supplementary file 15 — Figure EV4 Source Data [file 44321_2025_303_MOESM15_ESM.zip › Figure EV4/EV4A/EV4_L2_SMA+SMNC3(P8)+MW150(P0).tif]

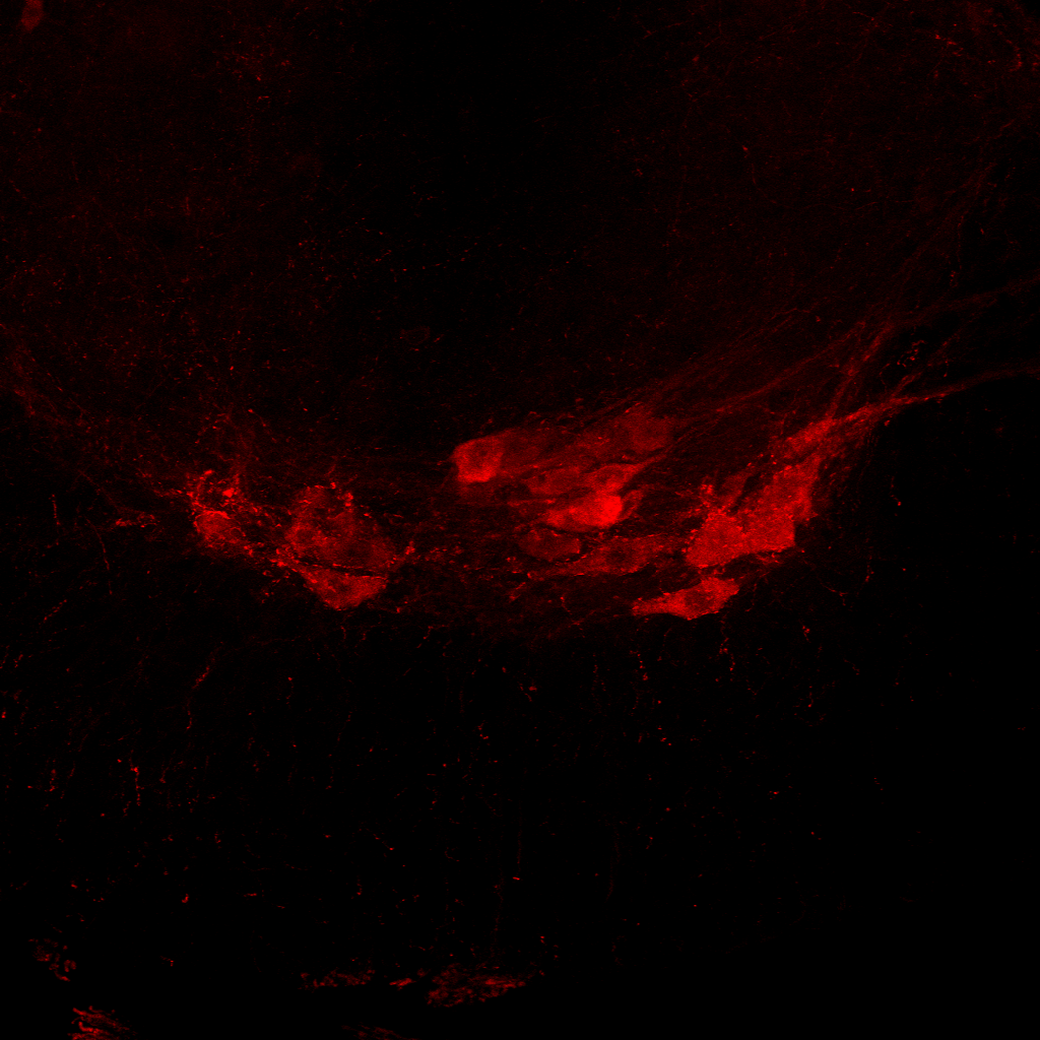

Supplement: Supplementary file 15 — Figure EV4 Source Data [file 44321_2025_303_MOESM15_ESM.zip › Figure EV4/EV4A/EV4-L2_SMA+SMNC3(P0).tif]

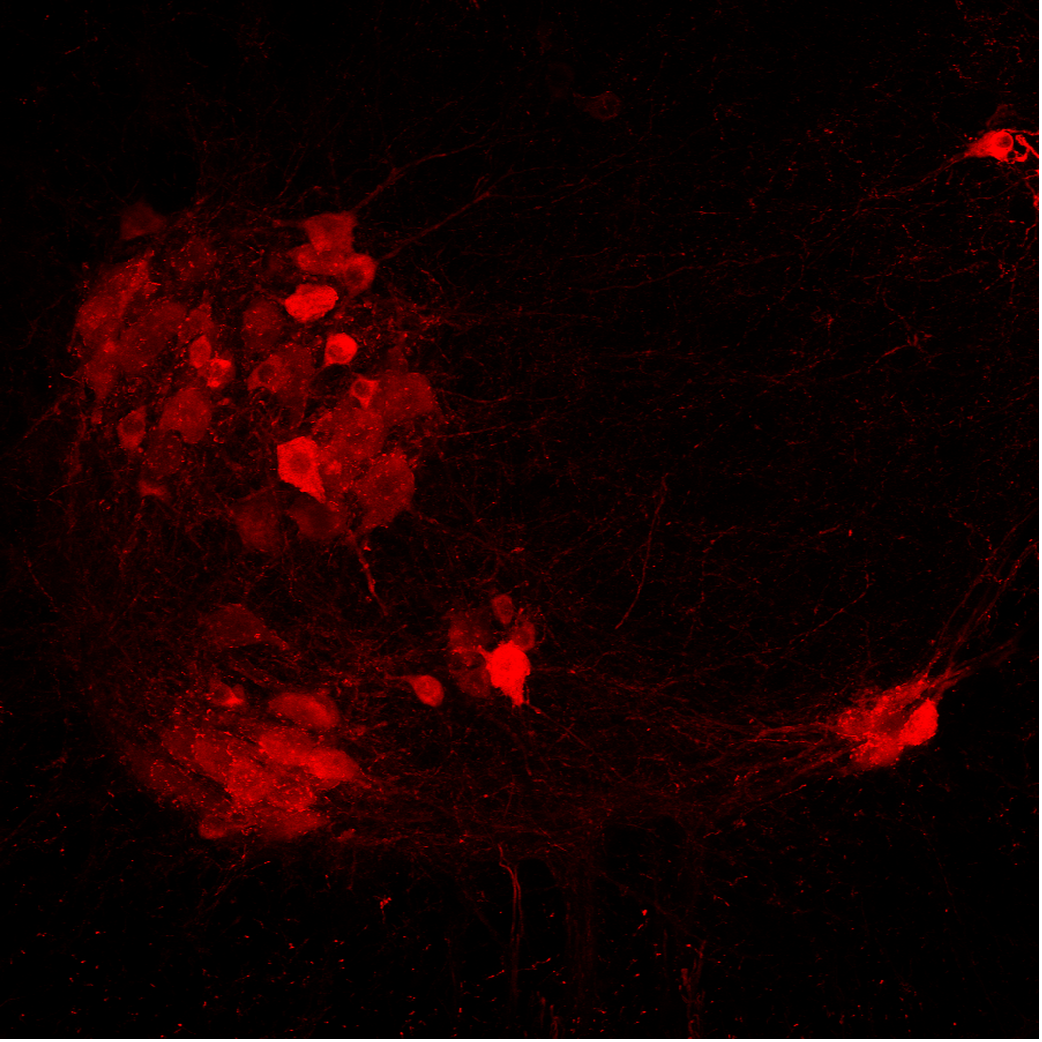

Supplement: Supplementary file 15 — Figure EV4 Source Data [file 44321_2025_303_MOESM15_ESM.zip › Figure EV4/EV4A/EV4_L5_SMA+SMNC3(P0).tif]

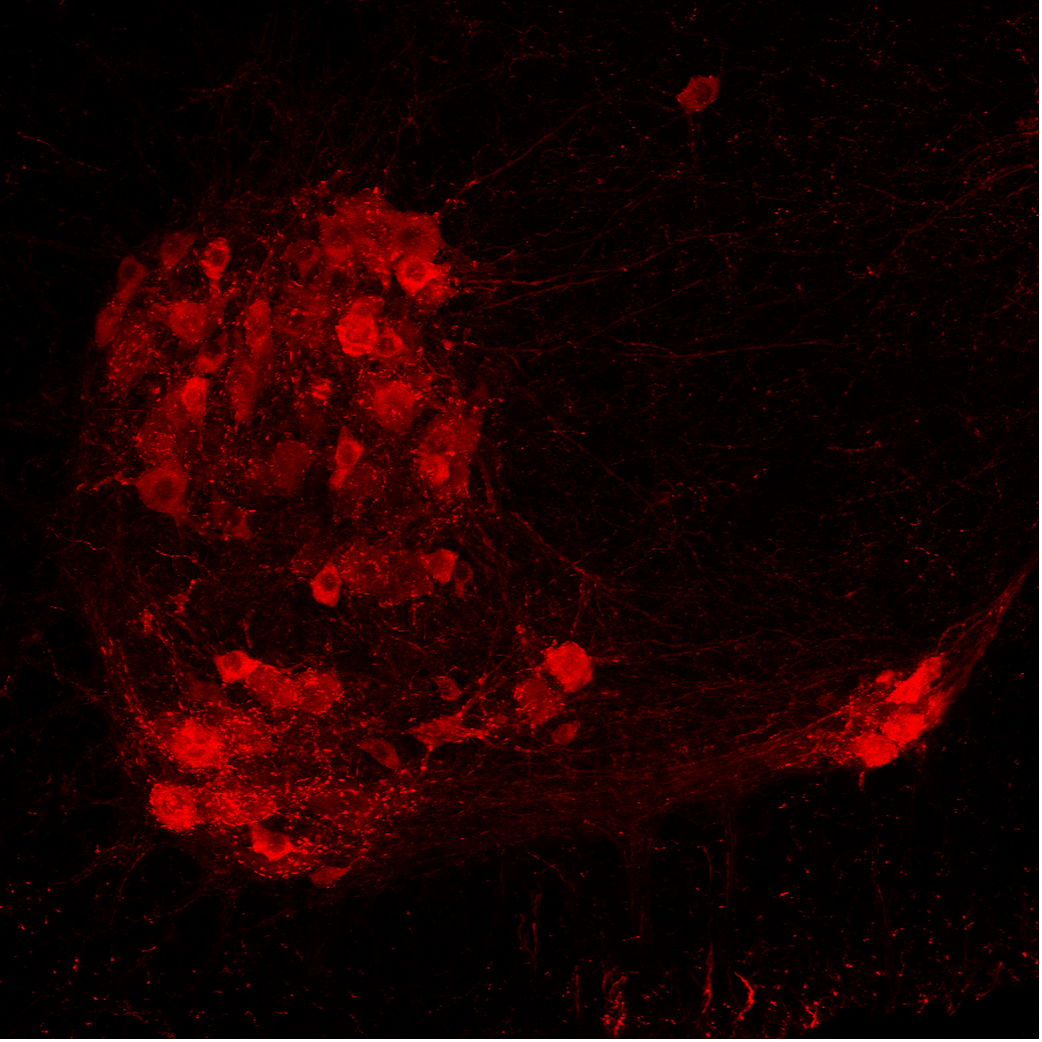

Supplement: Supplementary file 15 — Figure EV4 Source Data [file 44321_2025_303_MOESM15_ESM.zip › Figure EV4/EV4A/EV4_L5_WT.tif]

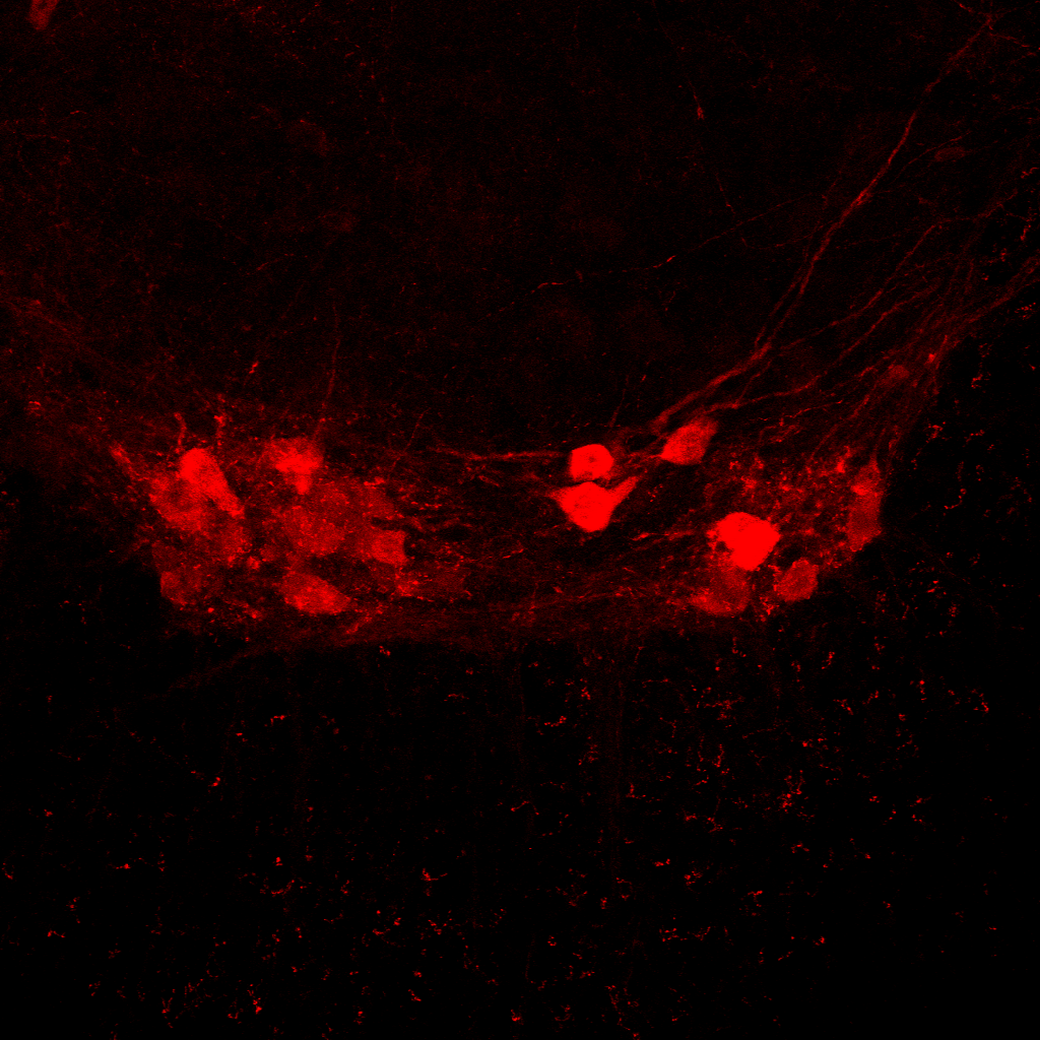

Supplement: Supplementary file 15 — Figure EV4 Source Data [file 44321_2025_303_MOESM15_ESM.zip › Figure EV4/EV4A/EV4_L2_WT.tif]

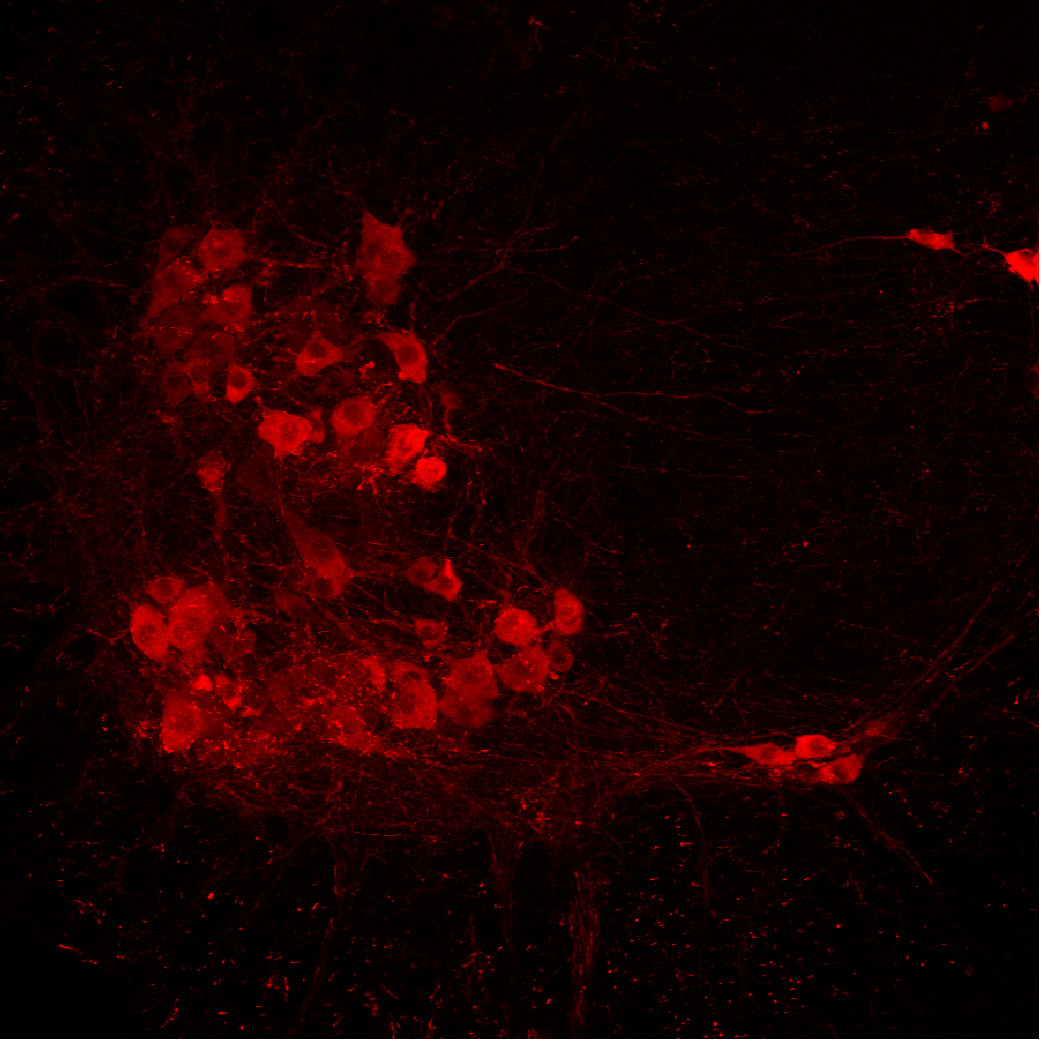

Supplement: Supplementary file 15 — Figure EV4 Source Data [file 44321_2025_303_MOESM15_ESM.zip › Figure EV4/EV4A/EV4_L5_SMA+SMNC3(P8)+MW150(P0).tif]
