# Supplementary material for: Identification of p38 MAPK inhibition as a neuroprotective strategy for combinatorial SMA therapy
Source: EMBO Mol Med. 2025 Sep 8;17(10):2762–86. doi: 10.1038/s44321-025-00303-6 (PMC12514318; doi:10.1038/s44321-025-00303-6)
Supplement: Supplementary file 16 — Expanded View Figures [file 44321_2025_303_MOESM16_ESM.pdf]

Expanded View Figures

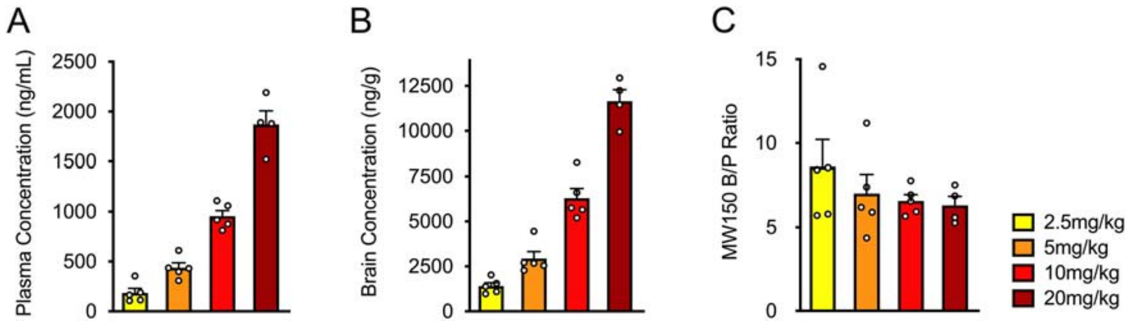

Figure EV1. Analysis of MW150 biodistribution in plasma and brain of SMA mice.

(A–C) MW150 concentration in plasma (**A**) and brain (**B**) and brain-to-plasma ratio (**C**) 3 h after a single IP injection of the indicated doses of MW150 in SMA mice at P10. Mean, SEM, and individual values from independent biological replicates (mice) for MW150 doses of 2.5 mg/kg ($n = 5$), 5 mg/kg ($n = 5$), 10 mg/kg ($n = 5$), and 20 mg/kg ($n = 4$) are shown. Source data are available online for this figure.

 © The Author(s)

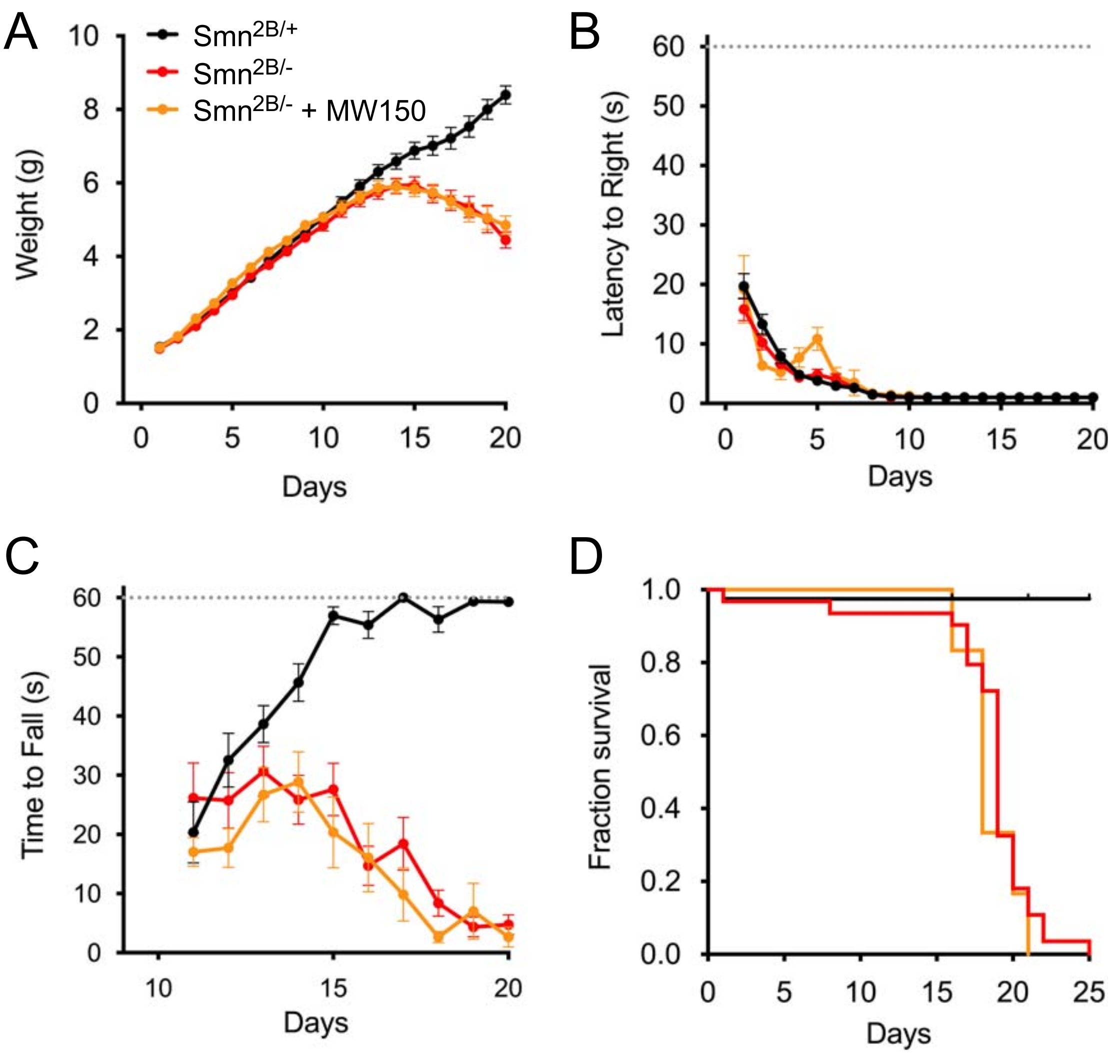

Figure EV2. MW150 treatment does not improve the SMA phenotype in *Smn*$^{2B/-}$ mice.

(**A**) Body weight of control *Smn*$^{2B/+}$ ($n = 32$) mice and *Smn*$^{2B/-}$ SMA mice either untreated ($n = 31$) or treated daily with MW150 (5 mg/kg) from P1 onward ($n = 12$). Data represent mean and SEM. Mixed-effects model ANOVA comparison of weight gain between *Smn*$^{2B/-}$ and *Smn*$^{2B/-}$ + MW150 mice: not significant. (**B**) Righting time from the same experimental groups shown in (**A**). Data represent mean and SEM. Mixed-effects model ANOVA comparison of righting time between *Smn*$^{2B/-}$ and *Smn*$^{2B/-}$ + MW150 mice: not significant. (**C**) Time to fall in the hindlimb suspension test from the same experimental groups shown in (**A**). Data represent mean and SEM. Mixed-effects model ANOVA comparison of hindlimb suspension test between *Smn*$^{2B/-}$ and *Smn*$^{2B/-}$ + MW150 mice: not significant. (**D**) Kaplan–Meier survival curves from the same experimental groups as in (**A**). Log-rank (Mantel–Cox) comparison of survival between *Smn*$^{2B/-}$ and *Smn*$^{2B/-}$ + MW150 mice: not significant. The data for untreated *Smn*$^{2B/+}$ and *Smn*$^{2B/-}$ mice are from a previously published study (Carlini et al, 2022). Source data are available online for this figure.

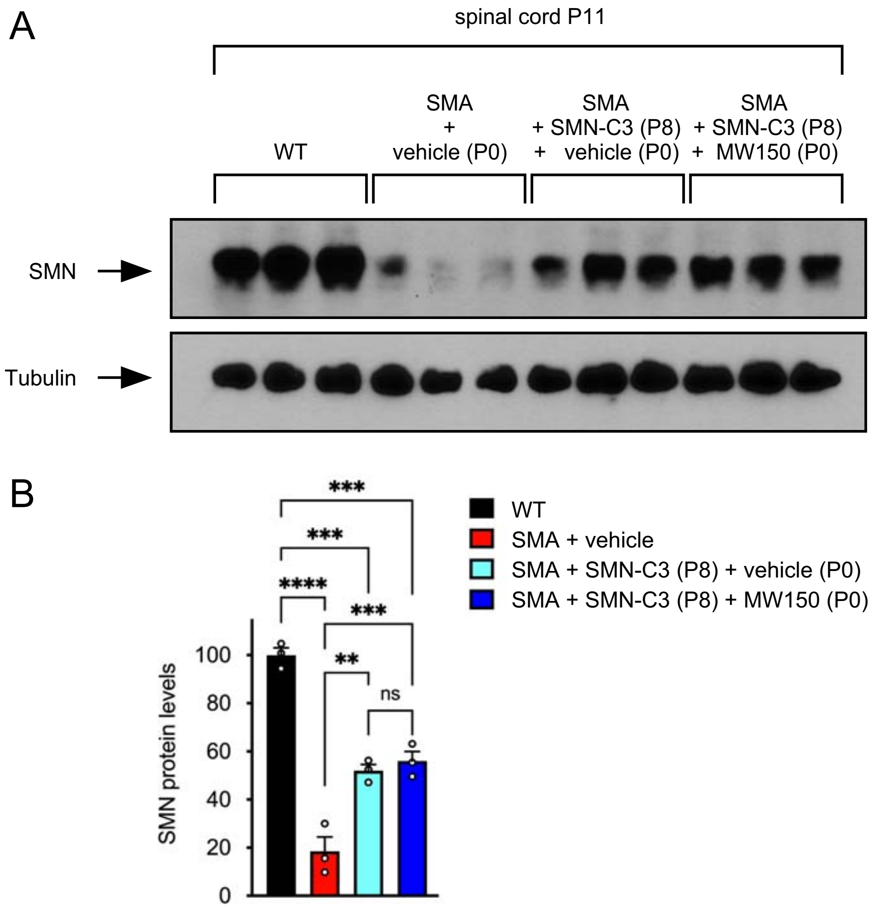

Figure EV3. MW150 does not increase SMN expression beyond the levels induced by delayed treatment with SMN-C3 in the spinal cord of SMA mice.

(**A**) Western blot analysis of SMN levels in P11 spinal cords from WT mice and SMA mice treated daily with vehicle or MW150 (5 mg/kg) starting at P0 and SMN-C3 (3 mg/kg) starting at P8 as indicated. Tubulin was used as loading control. (**B**) Quantification of SMN levels from the experiment in (**A**). Normalized mean, SEM, and individual values from three independent biological replicates are shown. One-way ANOVA and Tukey's post hoc test. $P < 0.0001$ (WT vs SMA+vehicle); $P = 0.0002$ (WT vs SMA + SMN-C3(P8) + vehicle(P0)); $P = 0.0003$ (WT vs SMA + SMN-C3(P8) + MW150(P0)); $P = 0.0019$ (SMA+vehicle vs SMA + SMN-C3(P8)+vehicle(P0)); $P = 0.0009$ (SMA + vehicle vs SMA + SMN-C3(P8) + MW150(P0)); ns not significant (SMA + SMN-C3(P8) + vehicle(P0) vs SMA + SMN-C3(P8) + MW150(P0)). Source data are available online for this figure.

© The Author(s)

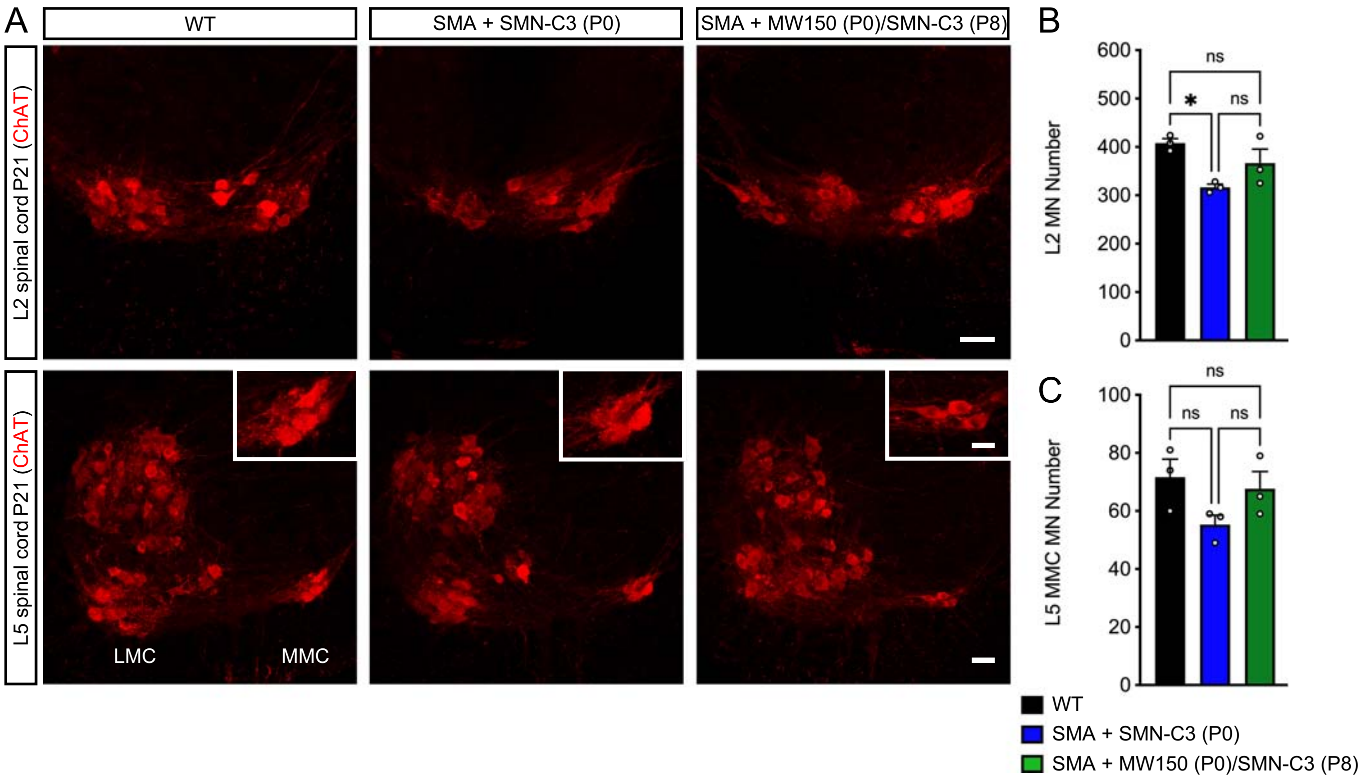

Figure EV4. Analysis of motor neuron survival in combinatorially treated adult SMA mice.

(**A**) ChAT immunostaining of L2 and L5 spinal cords isolated at P21 from uninjected WT mice and SMA mice injected daily with SMN-C3 (3 mg/kg) starting at P0 or injected with SMN-C3 (3 mg/kg) at P8 in combination with MW150 (5 mg/kg) starting at P0. L5 LMC and MMC motor neuron pools are indicated, and magnified views of L5 MMC motor neurons are shown in the insets. Scale bars = 50 and 25 μm (insets). (**B**) Total number of L2 motor neurons in the same experimental groups as in (**A**). Normalized mean, SEM, and individual values from three mice per experimental group are shown. One-way ANOVA with Tukey's post hoc test. $P = 0.0259$ (WT vs SMA + SMN-C3(P0); ns, not significant. (**C**) Total number of L5 MMC motor neurons in the same experimental groups as in (**A**). Normalized mean, SEM, and individual values from three mice per experimental group are shown. One-way ANOVA with Tukey's post hoc test. ns, not significant. Source data are available online for this figure.
